# Replicable in vivo physiological and behavioral phenotypes of the *Shank3B* null mutant mouse model of autism

**DOI:** 10.1186/s13229-017-0142-z

**Published:** 2017-06-15

**Authors:** Sameer C. Dhamne, Jill L. Silverman, Chloe E. Super, Stephen H. T. Lammers, Mustafa Q. Hameed, Meera E. Modi, Nycole A. Copping, Michael C. Pride, Daniel G. Smith, Alexander Rotenberg, Jacqueline N. Crawley, Mustafa Sahin

**Affiliations:** 1 F.M. Kirby Neurobiology Center, Translational Neuroscience Center, Department of Neurology, Boston Children’s Hospital, Harvard Medical School, Boston, MA 02115 USA; 2 MIND Institute, Department of Psychiatry and Behavioral Sciences, University of California Davis School of Medicine, Sacramento, CA 95821 USA; 3 Autism Speaks, Inc., Boston, MA USA; 4 Present address: BlackThorn Therapeutics, Inc., Cambridge, MA USA

**Keywords:** *Shank3B*, Pentylenetetrazol, Gamma oscillations, Repetitive behavior, Social behavior, Autism, Anxiety

## Abstract

**Background:**

Autism spectrum disorder (ASD) is a clinically and biologically heterogeneous condition characterized by social, repetitive, and sensory behavioral abnormalities. No treatments are approved for the core diagnostic symptoms of ASD. To enable the earliest stages of therapeutic discovery and development for ASD, robust and reproducible behavioral phenotypes and biological markers are essential to establish in preclinical animal models. The goal of this study was to identify electroencephalographic (EEG) and behavioral phenotypes that are replicable between independent cohorts in a mouse model of ASD. The larger goal of our strategy is to empower the preclinical biomedical ASD research field by generating robust and reproducible behavioral and physiological phenotypes in animal models of ASD, for the characterization of mechanistic underpinnings of ASD-relevant phenotypes, and to ensure reliability for the discovery of novel therapeutics. Genetic disruption of the *SHANK3* gene, a scaffolding protein involved in the stability of the postsynaptic density in excitatory synapses, is thought to be responsible for a relatively large number of cases of ASD. Therefore, we have thoroughly characterized the robustness of ASD-relevant behavioral phenotypes in two cohorts, and for the first time quantified translational EEG activity in *Shank3B* null mutant mice.

**Methods:**

In vivo physiology and behavioral assays were conducted in two independently bred and tested full cohorts of *Shank3B* null mutant (*Shank3B* KO) and wildtype littermate control (WT) mice. EEG was recorded via wireless implanted telemeters for 7 days of baseline followed by 20 min of recording following pentylenetetrazol (PTZ) challenge. Behaviors relevant to the diagnostic and associated symptoms of ASD were tested on a battery of established behavioral tests. Assays were designed to reproduce and expand on the original behavioral characterization of *Shank3B* KO mice. Two or more corroborative tests were conducted within each behavioral domain, including social, repetitive, cognitive, anxiety-related, sensory, and motor categories of assays.

**Results:**

Relative to WT mice, *Shank3B* KO mice displayed a dramatic resistance to PTZ seizure induction and an enhancement of gamma band oscillatory EEG activity indicative of enhanced inhibitory tone. These findings replicated in two separate cohorts. Behaviorally, *Shank3B KO* mice exhibited repetitive grooming, deficits in aspects of reciprocal social interactions and vocalizations, and reduced open field activity, as well as variable deficits in sensory responses, anxiety-related behaviors, learning and memory.

**Conclusions:**

Robust animal models and quantitative, replicable biomarkers of neural dysfunction are needed to decrease risk and enable successful drug discovery and development for ASD and other neurodevelopmental disorders. Complementary to the replicated behavioral phenotypes of the *Shank3B* mutant mouse is the new identification of a robust, translational in vivo neurophysiological phenotype. Our findings provide strong evidence for robustness and replicability of key translational phenotypes in *Shank3B* mutant mice and support the usefulness of this mouse model of ASD for therapeutic discovery.

**Electronic supplementary material:**

The online version of this article (doi:10.1186/s13229-017-0142-z) contains supplementary material, which is available to authorized users.

## Background

Since the initial discovery by Thomas Bourgeron and coworkers of *SHANK3* mutations in three cases of autism spectrum disorder (ASD) in 2007, many more cases have been reported [1–14]. *SHANK3* deficiency causes a monogenic form of ASD with a frequency of 0.5–1% of ASD cases [7]. Deletion in the *SHANK3* gene is also central to the cause of the rare neurodevelopmental disorder, Phelan McDermid Syndrome (PMS)[9]. The prevalence of *SHANK3* mutations has motivated the use of animal models with corresponding *Shank3* mutations to understand the underlying pathophysiology in cases of ASD, which harbor a *SHANK3* mutation, cases of PMS, and idiopathic ASD more broadly, with the goal of developing targeted pharmacological therapies.

Shank3, a scaffolding protein involved in the strengthening and stabilizing of synapses, is expressed in postsynaptic densities, a site of functional convergence of many ASD-related genes, rendering *Shank3* mutation a representative model of synaptopathy in ASD. A variety of mouse models have been generated with mutations in the *Shank3* gene, which include exon deletions affecting the ankyrin domain (*Shank3A*, [15–18], PDZ domain (*Shank3B*, [16, 19]), Homer domain (*Shank3ΔC*, [20]), and complete knockout of all isoforms [21]. Reduced social behaviors, elevated repetitive behaviors, cognitive impairments, abnormalities in dendritic spines, and aberrant in vitro electrophysiological measures of synaptic plasticity have been reported to various degrees in these models [15–17, 20–29]. Independent replications of these original reports, to confirm the strength of the various findings when conducted by other laboratories, have been conducted in only some cases. Further, to fully utilize these models for the development of novel therapies to treat ASD and PMS, quantitative and replicable biomarkers of neural dysfunction are needed in *Shank3* mutant mouse models.

Robustness and reproducibility of ASD-relevant phenotypes is essential to establish before an animal model can be effectively employed as a preclinical tool for therapeutic discovery. We therefore quantified seizure susceptibility and EEG power in the gamma frequency band in two cohorts of *Shank3B* null mutant mice. To evaluate the reproducibility of the previously reported social deficits and repetitive behaviors of this *Shank3B* mutant line [16], we investigated a wide range of behavioral phenotypes in two independent cohorts of *Shank3B* mice and their WT littermate controls. Behavioral testing followed a protocol of standardized methods and a precise order of testing at specified ages, and employed two or more corroborative tests within each behavioral domain, representing the rigorous experimental design developed by our collaborative Autism Speaks Preclinical Autism Consortium for Therapeutics (PACT).

EEG abnormalities, including seizures and subclinical epileptiform activity, are prevalent in both PMS and idiopathic ASD, consistent with the hypothesis that excitatory-inhibitory balance is widely disrupted in ASD [30–32]. Importantly, EEG can be similarly measured in both rodent models and in human patients and thus EEG phenotypes have great translational relevance [33]. To evaluate the utility of EEG as a quantitative biomarker, we characterized seizure propensity and oscillatory activity in the *Shank3B* mutant mice. The stability of the EEG phenotype was assessed in two independent cohorts to evaluate reproducibility of the phenotype.

*Shank3B* mutation resulted in a dramatic resistance to seizure induction and an enhancement of gamma band oscillatory activity indicative of enhanced inhibitory tone, in both mouse cohorts. Behavioral phenotypes including elevated levels of repetitive self-grooming and parameters of male-female reciprocal social interactions were replicated in both *Shank3B* cohorts. Thus, demonstration of the replicability of behavioral phenotypes and the identification of a novel, translational EEG phenotype in the commercially available *Shank3B* line from The Jackson Laboratory (JAX) provides evidence of a stable model that can be utilized consistently and reliably across independent laboratories. Detailed methods are provided for the generation of both the behavioral and the electrophysiological phenotypes, to enable the use of this model for both mechanistic and treatment studies broadly within the field.

## Methods

### Animals

Heterozygous breeding pairs of *Shank3B* mice (Shank3^tm2Gfng^, catalogue #017688) were obtained from The Jackson Laboratory (JAX) Repository, Bar Harbor, Maine, USA. This line, in which the *Shank3* mutation is at the PDZ site, was originally generated by Guoping Feng and colleagues at Duke University [16], and is maintained at JAX on a C57BL/6J background. Breeding colonies were independently developed at Boston Children’s Hospital, Boston, Massachusetts and the University of California Davis MIND Institute in Sacramento, California. *Shank3B* WT and null mutant mice were generated by crossing 8 to 14-week-old heterozygous males with age-matched heterozygous females. Genotyping was conducted as previously described [16]. In consideration of the low yield of the *Shank3B* breeding colony, all surviving offspring were used for testing. Both male and female mice were tested on behavioral assays. EEG analysis included only male mice. At both locations, all animals were housed in a temperature-controlled vivarium maintained on a 12-h light/dark cycle. All procedures were approved by the Animal Care and Use Committee at Boston Children’s Hospital (Boston, MA) and the Institutional Animal Care and Use Committee at the University of California Davis (Sacramento, CA), and were conducted in accordance with the National Institutes of Health Guide for the Care and Use of Laboratory Animals.

### In vivo electrophysiology (Sahin Laboratory, Boston)

#### Telemetry unit implantation

Cohort 1 consisted of seven male wildtype (WT) and eight male *Shank3B* null mutant (KO) mice; cohort 2 consisted of seven male WT and eight male *Shank3B* KO mice. Both cohorts were anesthetized with 100 mg/kg ketamine (Putney Vet, Portland, ME) and 10 mg/kg xylazine (Lloyd Inc, Shenandoah, IA) delivered via intraperitoneal (i.p.) injection. The mice were then intraperitoneally implanted with wireless telemetry transmitters (PhysioTel ETA-F10; DSI, Data Sciences International, St. Paul, MN) by threading the electrodes subcutaneously to the cranial cavity. Two burr holes, 1 mm in diameter, were drilled over the right olfactory bulb and left occipital lobe, into which the telemetry unit’s electrodes, connected to the leads of the transmitter, were placed epidurally and secured with stainless steel skull screws. Once in place, the skull screws were covered with dental cement (Dentsply International Inc., Milford, DE). Animals were subcutaneously injected at 0 and 24 h post-operation with 5 mg/kg meloxicam (Norbrook Laboratories, Newry, Northern Ireland) for analgesia. After 1 week of recovery, animals were individually housed in transparent home cages in a 12-h light/12 h dark, temperature, and humidity-controlled chamber with ad libitum access to food and water.

#### Data acquisition and seizure induction

One-channel video-EEG was recorded differentially between the reference (right olfactory bulb) and active (left occipital lobe) electrodes. Baseline data were continuously acquired over a period of 8 days, which included day and night cycles. Along with EEG sampled at 1000 Hz, the implanted transmitters also continuously measured core-body temperature at 200 Hz and locomotor activity at a sampling rate of 200 Hz.

All mouse cages were assigned to respective PhysioTel RPC receiver plates that transmitted data in real time from telemetry transmitters to a computer via the data exchange matrix using Dataquest ART software (Data Sciences International, St. Paul, MN). The recording and seizure induction times were standardized for all groups, and the high definition videos (30 frames/sec) were time-registered with the EEG.

At the end of baseline EEG acquisition, all animals were provoked with a convulsive dose (40 mg/kg; i.p.) of pentylenetetrazol (PTZ; Sigma-Aldrich, Co., St. Louis, MO), a GABA_A_ receptor antagonist, to measure seizure susceptibility. Historically in our laboratory, this dose has been sufficient to induce seizure in more than 50% of healthy rodents [34, 35].

### Data analysis

The first 24 h of baseline EEG recordings were considered an acclimation period and were discounted from analysis. The electrophysiological data presented here are derived from 168 h of subsequent video-EEG followed by 20 min of post-PTZ recordings. Mice were continuously monitored for clinical and electrographic epileptic activity during both periods.

#### Video EEG

Following PTZ administration, mice were monitored for 20 min for any signs of observable epileptic activity and post hoc verified by the blinded review of the video EEG. Latency and frequency of PTZ-provoked seizures were used as measures of seizure susceptibility. Latency to myoclonic seizures was defined as the time from injection to the first visible myoclonus, indicative of the onset of epileptic activity. However, for statistical purposes, we assigned latency to 1200 s in mice that did not have a seizure during the 20 min of monitoring.

Power in frequency bands of the baseline EEG was calculated by transforming the raw EEG signal to frequency domain using the fast fourier transform (FFT) technique. The power in the gamma frequency band (30–80 Hz) was expressed as a ratio of its absolute power to the total absolute power (1–80 Hz), to compensate for inter-subject variability and artifacts.

The video-EEG was scored offline for behavioral and electrographic seizures. An epileptiform discharge was defined as a run of continuous spikes ≥ 5 s in duration on the EEG. The epileptiform discharges were counted by optimizing the automated seizure detection algorithm in Neuroscore (Data Sciences International, St. Paul, MN)[35]. Individual spike characteristics such as amplitude, duration, frequency, and inter-spike intervals were used to differentiate the epileptiform spikes from the baseline spikes or electrical and mechanical artifacts. Automatically detected events were verified by visual inspection against the real-time video and spectrogram.

#### Circadian biometrics

Core body temperature and locomotor activity were also continuously sampled at 200 Hz for 8 days with EEG. The intraperitoneal placement of telemetry transmitter unit enables the measurement of core body temperature in these recordings. Actigraphy measures locomotor activity of the mouse inside the home cage. Activity counts were summed over 7 days for analysis.

#### Statistics

Data were analyzed using GraphPad Prism (v 6, GraphPad Software Inc., La Jolla, CA) with significance level defined at *p* < 0.05. All results are presented as mean ± SEM. Student’s unpaired *t* tests were used to compare activity counts, seizures, and power in the EEG frequency bands. A log-rank (Mantel-Cox) test was performed to compare the Kaplan–Meier analysis for seizure incidence rate and latency.

### Behavioral assays (Crawley Laboratory, Sacramento)

Cohort 1 consisted of 12 male WT littermates (WT), 12 female WT, 12 male *Shank3B* null mutants (KO), and 10 female *Shank3B* KO. Cohort 2 consisted of *N* = 12 male WT, *N* = 10 female WT, *N* = 9 male *Shank3B* KO, and *N* = 12 female *Shank3B* KO. Testing was performed during the light phase of the circadian cycle. Mice were tested at the ages shown and in the sequence listed in Additional file 1: Table S1. Assays of high relevance to the diagnostic symptoms of ASD were conducted in both cohorts 1 and 2. In some cases, assays relevant only to associated symptoms of ASD, which showed normal phenotypes in *Shank3B* KO in cohort 1, were not repeated in cohort 2. Order of testing was determined by the longitudinal juvenile and adult ages required for some tests, and by the principle of conducting the most stressful tests last. For all behavioral assays, procedures were employed that are consistent with best practices from the behavioral neuroscience literature, and from our previous publications [24, 36–50]. For all non-automated assays, videos were scored by investigators uninformed of genotype.

#### Juvenile reciprocal social interactions

Juvenile reciprocal social interactions were tested in mice between postnatal days 24–26 (Additional file 1: Table S1) in the Noldus PhenoTyper Observer 3000 chamber (25× 25 × 35 cm), as previously described. The floor of the arena was covered with a 0.5-cm layer of clean bedding. Subjects and stimulus partners were individually housed in a clean cage for 1 h before the test. An individual subject mouse was then placed in the arena, with an age- and sex-matched juvenile WT partner. Interactions were recorded for 10 min, the period during which the majority of the social interactions occur. Parameters of juvenile mouse social behaviors were chosen from the established literature and from our previous studies [38, 44, 48].

#### Elevated plus-maze

Elevated plus-maze anxiety-related testing was performed according to previously described procedures [47, 51] using a standard mouse apparatus (Med Associates, St. Albans, VT). The maze had two open arms (35.5 × 6 cm) and two closed arms (35.5 × 6 cm) radiating from a central area (6 × 6 cm). A 0.5-cm-high lip surrounded the edges of the open arms. 20-cm-high walls enclosed the closed arms. The apparatus was cleaned with 70% ethanol before the beginning of the first test session and after each subject mouse. Room illumination was ~ 300 lux.

#### Light↔dark transitions

Light↔dark anxiety-related exploration was measured according to previously published procedures [47, 51]. Subject mice were placed in the brightly illuminated, large chamber (~400 lux). The smaller dark chamber (~5 lux) was entered by traversing a partition between the two chambers. Subject mice freely explored for 10 min. Time in the dark side chamber and total number of transitions between the light and dark side chambers were automatically recorded using LabVIEW 8.5.1 software (National Instruments, Austin, TX, developed by George Dold, Research Services Branch, National Institute of Mental Health, Bethesda, MD). Room illumination was ~ 400 lux.

#### Open field locomotion

General exploratory locomotion in a novel open field environment was assayed for 30 min using Versamax Accuscan videotracking, as previously described [47, 50]. Open field activity was considered an essential control for effects on physical activity, e.g., sedation or hyperactivity, which could confound the interpretation of results from the reciprocal interactions, self-grooming, fear conditioning, and social approach tasks. The testing room was illuminated at ~40 lux.

#### Novel object recognition

The novel object recognition test was conducted in opaque matte white (P95 White, Tap Plastics, Sacramento, CA) open field arenas (40 × 60 × 23 cm), using methods similar to those previously described [24, 49]. The experiment consisted of three sessions: a 30-min exposure to the open field arena, a 10-min familiarization session, and a 5-min recognition test. On day 1, each subject was habituated to a clean empty open field arena for 30 min. 24 h later, each subject was returned to the open field arena for the habituation phase, for 10 min. The mouse was then removed from the open field and was placed in a clean temporary holding cage for approximately 2 min. Two identical objects were placed in the arena. Each subject was returned to the open field in which it had been habituated and was allowed to freely explore for 10 min. After the familiarization session, subjects were returned to their holding cages, which were transferred from the testing room to a nearby holding area. The open field was cleaned with 70% ethanol and let dry. One clean familiar object and one clean novel object were placed in the arena, where the two identical objects had been located during in the familiarization phase. 1 h after the end of the familiarization session, each subject was returned to its open field for a 5-min recognition test, during which time it was allowed to freely explore the familiar object and the novel object. The familiarization session and the recognition test were videotaped and scored with EthoVision XT videotracking software (version 9.0, Noldus Information Technologies, Leesburg, VA). Object investigation was defined as time spent sniffing the object when the nose was oriented toward the object and the nose-object distance was 2 cm or less. Recognition memory was defined as spending significantly more time sniffing the novel object than sniffing the familiar object. Total time spent sniffing both objects was used as a measure of general exploration. Time spent sniffing two identical objects during the familiarization phase confirmed the lack of an innate side bias. Objects utilized were plastic toys: a smooth plastic orange cone and a hard-plastic green cylinder with ribbed sides.

#### Acoustic startle threshold and prepulse inhibition of acoustic startle

Acoustic startle and prepulse inhibition of acoustic startle were measured using the SR-Laboratory System (San Diego Instruments, San Diego, CA) as described previously [39, 46, 51]. Test sessions began by placing the mouse in the Plexiglas restrainer for a 5-min acclimation period. For the next 8 min, mice were presented with each of 6 trial types across six discrete blocks of trials, for a total of 36 trials. The intertrial interval was 10–20 s. One trial type measured the response to no stimulus (baseline movement). The other five trial types measured startle responses to 40 ms sound bursts of 80, 90, 100, 110, or 120 dB. The six trial types were presented in pseudorandom order such that each trial type was presented once within a block of six trials. Startle amplitude was measured every 1 ms over a 65 ms period beginning at the onset of the startle stimulus. The maximum startle amplitude over this sampling period was taken as the dependent variable. Background noise level of 70 dB was maintained over the duration of the test session. For prepulse inhibition of acoustic startle, mice were presented with each of seven trial types across six discrete blocks of trials for a total of 42 trials, over 10.5 min. The inter-trial interval was 10–20 s. One trial type measured the response to no stimulus (baseline movement) and another measured the startle response to a 40 ms 110 dB sound burst. The other five trial types were acoustic prepulse stimulus plus acoustic startle stimulus trials. The seven trial types were presented in pseudorandom order such that each trial type was presented once within a block of seven trials. Prepulse stimuli were 20 ms tones of 74, 78, 82, 86, and 92 dB intensity, presented 100 ms prior to the 110 dB startle stimulus. Startle amplitude was measured every 1 ms over a 65 ms period, beginning at the onset of the startle stimulus. The maximum startle amplitude over this sampling period was taken as the dependent variable. A background noise level of 70 dB was maintained over the duration of the test session.

#### Repetitive self-grooming and marble burying

Spontaneous repetitive self-grooming behavior was scored as previously described [24, 38, 43]. Each mouse was placed individually into a standard mouse cage, (46 cm length × 23.5 cm wide × 20 cm high). Cages were empty to eliminate digging in the bedding, which is a potentially competing behavior. The room was illuminated at ~ 40 lux. A front-mounted CCTV camera (Security Cameras Direct) was placed at ~ 1 m from the cages to record the sessions. Sessions were videotaped for 20 min. The first 10-min period was habituation and was unscored. Each subject was scored for cumulative time spent grooming all the body regions during the second 10 min of the test session.

Marble burying was conducted in a mouse cage containing bedding at a depth of 2 cm. 20 black glass marbles were arranged in a 4 × 5 cm grid on top of the bedding. The mouse was placed in the center of the cage for a 30-min exploration period, under 15 lux illumination. Number of marbles at least 50% covered by bedding were scored as buried.

#### Three-chambered social approach

Social approach was tested in a modified automated three-chambered apparatus using methods similar to those previously described [38, 43, 51–54]. Noldus EthoVision XT videotracking software (version 9.0, Noldus Information Technologies, Leesburg, VA) was employed to increase throughput. The updated apparatus was a rectangular three-chambered box, 40 × 60 × 23 cm, fabricated from matte white acrylic (P95 White, Tap Plastics, Sacramento, CA). Opaque retractable doors (12 × 33 cm) were designed to create optimal entryways between chambers (5 × 10 cm), while providing maximal division of compartments. Three zones, defined using the EthoVision XT software, detected time in each chamber for each phase of the assay. Zones to score sniffing were defined as the annulus extending 2 cm from each novel object or novel mouse enclosure (inverted wire cup, Galaxy stainless steel pencil and utility cup, Kitchen Plus, http:// www.kitchen-plus.com). Direction of the head, facing toward the cup enclosure, defined sniff time. A top mounted infrared-sensitive camera (Ikegami ICD-49, B&H Photo, New York, NY) was positioned directly above every two three-chambered units. Infrared lighting (Nightvisionexperts.com) provided uniform, low level illumination. The subject mouse was first contained in the center chamber for 10 min, then explored all three empty chambers during a 10-min habituation session, then explored the three chambers containing a novel object in one side chamber and a novel mouse in the other side chamber. Lack of innate side preference was confirmed during the initial 10 min of habituation to the entire arena. Novel stimulus mice were 129Sv/ImJ, a relatively inactive strain, aged 10–14 weeks old, and were matched to the subject mice by sex. Number of entries into the side chambers served as a within-task control for levels of general exploratory locomotion.

Three-chambered social approach, developed by our group in 2004 [36, 37], is a simple binary assay that determines yes or no sociability within genotype. In our extensive early development of this task, evaluating many control parameters, we determined that the absolute number of seconds spent interacting with the novel mouse varied considerably across repeated testing and did not represent a sensitive enough measure of sociability to quantitatively compare time with the novel mouse across genotypes or across treatment groups. Sociability in this assay is defined as more time in the chamber with the novel mouse than in the chamber with the novel object, and more time sniffing the novel mouse than sniffing the novel object, within each genotype or within each treatment group.

#### Olfactory habituation/dishabituation

Evaluation of responses to nonsocial and social odors was conducted as previously described [24, 40, 44]. Subjects were tested in a clean, empty standard mouse cage. Odor-saturated 6-in cotton-tipped applicators (Fisherbrand, ThermoFisher Scientific, Hudson, NH) were used to deliver odor stimuli. To reduce novelty-induced exploratory activities, each subject was first acclimated for 45 min in the empty testing cage containing one clean dry cotton applicator. The test consisted of 15 sequential 2-min trials: 3 presentations of plain tap water, 3 presentations of banana odor (prepared from imitation banana extract; McCormick; 1:100 dilution), 3 presentations of vanilla odor (prepared from vanilla extract; McCormick; 1:100 dilution), 3 presentations of social odor from social cage 1, 3 presentations of social odor from social cage 2. Water, banana, and vanilla olfactory stimuli were prepared by dipping the cotton tip briefly into the solution. Social olfactory stimuli were prepared by wiping a swab in a figure 8 motion across the bottom of a soiled cage of same-sex mice who were unfamiliar to the subject mice. For each subject, one soiled cage of 129/SvImJ mice and one soiled cage of B6 mice were the sources of the two distinct social odors. Time spent sniffing the swab was quantified from videos by a well-trained investigator, blind to genotype, using a stopwatch.

#### Hot plate

For the hot plate test, the mouse was placed on the arena surface, which was kept at a constant temperature of 55 ^o^C (IITC Life Science Inc., Woodland Hills, CA). Latency to first response, such as licking or shaking paws, was recorded. To prevent tissue damage, a cut-off latency of 30 s was applied.

#### Male-female social interaction

The male-female reciprocal social interaction test was conducted as previously described [24, 42, 46, 54]. Each freely moving male subject was paired for 5 min with a freely moving unfamiliar estrous WT female. Visual observation of vaginal swelling and color on a 1–3 scale was used to determine estrous state. A closed-circuit television camera (Panasonic, Secaucus, NJ, USA) was positioned at an angle from the Noldus PhenoTyper arena (Noldus, Leesburg, VA) for optimal video quality. An ultrasonic microphone (Avisoft UltraSoundGate condenser microphone capsule CM15; Avisoft Bioacoustics, Berlin, Germany) was mounted 20 cm above the cage. Sampling frequency for the microphone was 250 kHz, and the resolution was 16 b. The entire apparatus was contained in a sound-attenuating environmental chamber (Lafayette Instruments, Lafayette, IN) under red light illumination (~10 lux). Duration of nose-to-nose sniffing, nose-to-anogenital sniffing, and following were scored using Noldus Observer 8.0XT event recording software (Noldus, Leesburg, VA). Ultrasonic vocalization spectrograms were displayed using Avisoft software. Calls were quantified manually by a well-trained investigator blind to genotype.

#### Fear conditioning

Standard delay contextual and cued fear conditioning was conducted using an automated fear-conditioning chamber (Med Associates, St Albans, VT, USA) as previously described [44, 49]. The conditioning chamber (32 × 25 × 23 cm, Med Associates) was interfaced to a PC installed with VideoFreeze software (version 1.12.0.0, Med Associates) and was enclosed in a sound-attenuating cubicle. Training consisted of a 2-min acclimation period followed by three tone-shock (CS–US) pairings (80 dB tone, duration 30 s; 0.5 mA footshock, duration 1 s; intershock interval 90 s) and a 2.5-min period, during which no stimuli were presented. The environment was well lit (~100 lux), with a stainless steel grid floor and was swabbed with vanilla odor cue (prepared from vanilla extract; McCormick; 1:100 dilution). A 5-min test of contextual fear conditioning was performed 24 h after training, in the absence of the tone and footshock, but in the presence of 100 lux overhead lighting, vanilla odor, and chamber cues identical to those used on the training day. Cued fear conditioning was conducted 48 h after training, in a novel environment with distinct visual, tactile, and olfactory cues. Overhead lighting was turned off. The cued test consisted of a 3-min acclimation period followed by a 3-min presentation of the tone CS and a 90-s exploration period. Cumulative time spent freezing in each condition was quantified by VideoFreeze software (Med Associates).

#### Morris water maze acquisition

Spatial learning was assessed in the Morris water maze using procedures and equipment as previously described [24, 39, 44]. The apparatus was a circular pool (120 cm diameter) filled 45 cm deep with tap water rendered opaque with the addition of non-toxic white paint (Crayola, Easton, PA). Distal room cues were black and white cardboard patterns on the walls, approximately 1 m from the circumference of the pool. Trials were videotaped and scored with EthoVision XT videotracking software (version 9.0, Noldus Information Technologies, Leesburg, VA). Acquisition training consisted of 4 trials a day for 7 days. Each training trial began by lowering the mouse into the water close to the pool edge, in a quadrant that was either right of, left of, or opposite to, the target quadrant containing the platform. The start location for each trial was alternated in a semi-random order for each mouse. The hidden platform remained in the same quadrant for all trials during acquisition training for a given mouse, but varied across subject mice. Mice were allowed a maximum of 60 s to reach the platform. A mouse that failed to reach the platform in 60 s was guided to the platform by the experimenter, using a wire cage lid. Mice were left on the platform for 15 s before being removed. After each trial, the subject was placed in a cage lined with absorbent paper towels and was allowed to rest under an infrared heating lamp for 60 s. Acquisition training continued until the WT control group reached the criterion of 15-s latency to find the hidden platform. 3 h after the completion of training on the day criterion was met by the WT group, the platform was removed and all mice were tested in a 60-s probe trial, to confirm that their spatial learning had been acquired by using distal environmental room cues. Parameters recorded during training days were latency to reach the platform, total distance traveled, and swim speed. Time spent in each quadrant and number of crossings over the trained platform location and over analogous locations in the other quadrants was used to analyze probe trial performance. Reversal of water maze spatial location acquisition was not conducted, based on the results from the initial acquisition, as described below.

#### Statistics

Data were analyzed using GraphPad Prism (v 6, GraphPad Software Inc., La Jolla, CA) with significance level defined at *p* < 0.05. All results are presented as mean ± SEM, using statistical tests previously described [24, 44, 55]. Repeated measures analysis of variance (ANOVA) was used to compare genotypes for most of the behavioral assays. Bonferroni-Dunn post hoc analysis was conducted to compare individual groups in cases of a significant ANOVA value. Student’s unpaired *t* tests were used to compare genotypes on self-grooming, on parameters of juvenile and of male-female reciprocal social interactions and to compare time spent sniffing novel and familiar objects in novel object recognition.

## Results

### Electrophysiology

#### Shank3B knockouts do not exhibit spontaneous seizures

No spontaneous behavioral seizures or epileptiform abnormalities were observed in either cohort of *Shank3B* KO and WT controls during 168 h of baseline video-EEG recordings. Epileptiform discharges defined as runs of spikes ≥ 5 s found in the knockout groups were not significantly different from the spikes recorded in WT controls.

#### Shank3B KO mice are protected from PTZ-induced seizures

**Fig. 1:**
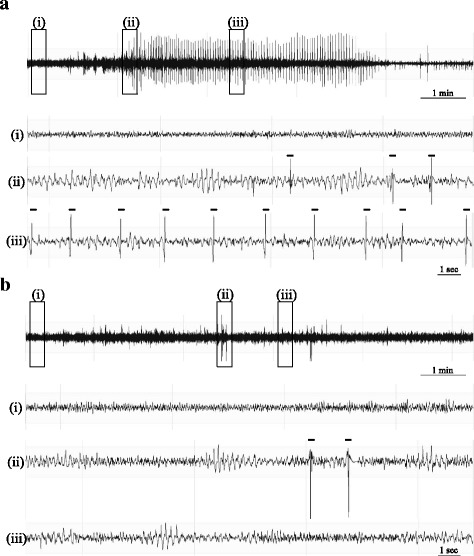
Representative EEG after PTZ injection. **a** Representative 10-min EEG from WT mouse shows three clinical epileptic stages after PTZ administration progressing from a healthy baseline (*i*), to developing epileptic spike trains (*ii*), and a run of frequent myoclonic seizures as indicated by markers (*iii*). **b** Representative 10-min EEG from *Shank3B* knockout exhibits epileptic spike-trains but has noticeably fewer myoclonic seizures than wildtypes

Post PTZ injection, mice exhibited various clinical epileptic stages from myoclonic jerking to behavioral arrest to generalized tonic-clonic seizure (GTCS) as represented in Fig. 1. The representative 10-min trace shows the gradual progression of a WT animal’s EEG from healthy baseline (i) to epileptic spikes (ii) and eventually frequent myoclonic seizures (iii) after PTZ injection (Fig. 1a). On the contrary, the video- EEG recorded from a *Shank3B* KO exhibits very few myoclonic seizures at a longer latency post injection (Fig. 1b). The marked events are indicative of digitally detected and visually verified myoclonic seizures.

**Fig. 2:**
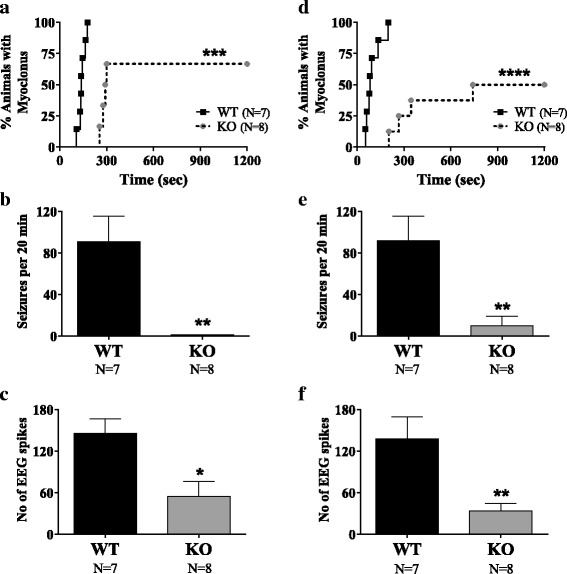
Analysis of PTZ-induced seizures in cohort 1 (**a**-**c**) and in cohort 2 (**d**-**f**). **a** and **d** Incidence and latency to first myoclonus after PTZ injection. Kaplan–Meier survival curve is used to display percentage incidence of first myoclonus (y-axis) and its latency (x-axis), after PTZ injection (40 mg/kg). Curve comparison in first cohort shows that the incidence of myoclonus rate of (100%) in WT group was significantly higher than in the *Shank3B* KO group (67%) in **a**, which was replicable in the second cohort with only 50% knockouts experiencing a myoclonus in comparison to 100% of WT mice in **d**. Moreover, the *Shank3B* KO mice had a significantly longer latency to the first myoclonic seizure relative to WT controls. **b** and **e** Myoclonic seizure count. The frequency of PTZ-induced myoclonic seizures per 20 min of recording was significantly reduced in *Shank3B* KO mice relative to WT as replicated in two separate cohorts (**b** for cohort 1 and **e** for cohort 2) . **c** ﻿and **f** Epileptic spike count. The number of epileptic spikes on the EEG was also significantly lower in *Shank3B* KO mice relative to WT mice in both cohorts (**c** for cohort 1 and **f** for cohort 2). *p < 0.05, **p < 0.01, ***p < 0.001, ****p < 0.0001

While 100% of the WT controls experienced a myoclonus, the percentage was relatively lower in *Shank3B* KO (67%) in the first cohort. Kaplan–Meier analysis (Fig. 2a) displaying the incidence of first myoclonus in both groups revealed a significant difference between the curves (Mantel-Cox test, chi-square = 12.64; *p* = 0.0004). The latency to first myoclonic seizure was significantly longer in the *Shank3B* KO mice relative to WT controls (Fig. 2a). This significant genotype difference was replicated in cohort 2 (chi-square = 16.36; *p* < 0.0001) with only 50% of the *Shank3B* KO mice having a myoclonus. *Shank3B* KO mice were strongly protected from PTZ seizures such that their shortest latency to myoclonus was considerably longer than all of WT mice.

While two of the WT mice from the first cohort and one in the second cohort progressed from myoclonic jerking to GTCS, none of the *Shank3B* KO mice exhibited a GTCS following PTZ injection. Frequency of myoclonic seizures and epileptic spikes were digitally scored (refer to methods for details). The total number of myoclonic seizures in *Shank3B* KO mice was significantly lower in comparison to WT controls (1 ± 0.3 vs 91 ± 25 seizures per 20 min, unpaired *t* test, *p* = 0.007) in the first cohort (Fig. 2b). Similarly, in the second cohort, *Shank3B* KO had significantly fewer seizures per 20 min of monitoring (10 ± 9 vs 92 ± 23, *p* = 0.004). Epileptic EEG spikes were observed in all animals of both groups upon PTZ administration. The frequency of these spikes (Fig. 2c) was again significantly lower in *Shank3B* KO relative to WT mice (54 ± 22 vs 145 ± 21, unpaired *t* test, *p* = 0.012) in the first cohort and again, very robustly reproducible in the second cohort (34 ± 11 vs 138 ± 32 spikes per 20 min, *p* = 0.006).

#### Gamma oscillations are increased in Shank3B KO mice

**Fig. 3:**
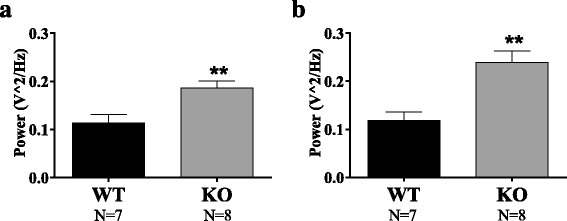
EEG gamma power. Spectral analysis of the 1-h pre-PTZ EEG shows higher power in the gamma frequency band (30–80 Hz). These results were replicable in both cohorts **p < 0.01. (**a** for cohort 1 and **b** for cohort 2)

Spectral power in the gamma (30–80 Hz) frequency band on EEG was calculated using the FFT technique. We performed spectral analysis in the 1-h baseline before PTZ. *Shank3B* KO in the first cohort exhibited increased power in the gamma frequency band as compared to WT (unpaired *t* test, *p* = 0.0073) as represented in Fig. 3. Similarly, gamma oscillations were heightened in *Shank3B* KO of cohort 2 relative to WT (*p* = 0.0017). The increase in gamma power on EEG suggests a plausible increase in GABAergic reserve in this phenotype that may account for the seizure resistance.

Spectral power in the low frequency bands (delta, theta, alpha, and beta) did not show any differences between KO and WT across both cohorts (Additional file 2: Figure S2).

#### Shank3B KO mice are hypoactive

**Fig. 4:**
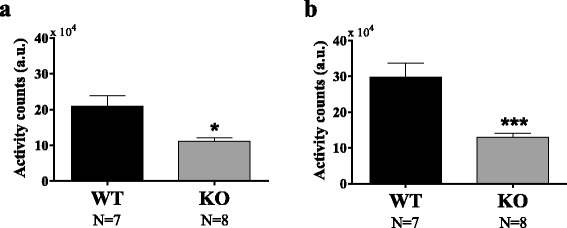
Locomotor activity at baseline. The total locomotor activity measured from 1 week baseline of male *Shank3B* knockout (KO) mice was significantly lower than wildtypes. The motor hypoactivity in KO as compared to WT was significant in both, cohort 1, *p < 0.05, and cohort 2, ***p < 0.001 (**a** for cohort 1 and **b** for cohort 2)

Actigraphy was recorded from all freely moving implanted animals. Total activity counts across 168 h of recording reveal that the *Shank3B* KO in the first cohort were significantly hypoactive (11.2 ± 0.94 vs 20.94 ± 2.97, arbitrary units) in comparison with the WT controls (unpaired *t* test, *p* = 0.014) (Fig. 4). The second cohort supported the finding and showed a stronger trend for hypomotor activity in the *Shank3B* KO relative to WT (12.93 ± 1.14 vs 19.82 ± 3.89, arbitrary units; *p* = 0.0007).

### Behavior

#### Shank3B KO display normal juvenile reciprocal social interactions

No deficits were detected in *Shank3B* KO on parameters of social interactions in same-sex dyads of juveniles. As shown in Additional file 1: Table S2, scores did not differ between genotypes on measures of cumulative time spent in nose-to-nose sniffing, number of bouts of nose-to-nose sniffing, cumulative time spent in nose-to-anogenital sniffing, number of bouts of nose-to-anogenital sniffing, cumulative time spent following, number of bouts of following, or number of bouts of front approach. Both males and females displayed normal juvenile reciprocal social interactions. Absence of genotype differences was seen in both cohorts 1 and 2.

#### Shank3B KO display mostly normal elevated plus-maze behavior

Anxiety-related parameters on the elevated plus-maze were normal for *Shank3B* KO in most cases. As shown in Additional file 1: Table S3, % open arm time did not differ between genotypes in either sex in either cohort 1 or cohort 2. Number of open arm entries did not differ between genotypes in males in either cohort 1 or cohort 2. Female *Shank3B* KO displayed higher numbers of open arm entries in cohort 2, indicating less anxiety-related behavior, although female genotypes did not differ on this measure in cohort 1. Total number of entries, the control measure for general exploration, was lower for *Shank3B* KO than WT in cohort 1, primarily in the males, but did not differ between genotypes in either sex for cohort 2.

#### Shank3B knockouts display anxiety-like behavior in the light↔dark exploration assay in cohort 1 but not in cohort 2

Anxiety-related parameters in the light↔dark chamber indicated anxiety-like behavior in both males and females in cohort 1 only. As shown in Additional file 1: Table S3, both male and female *Shank3B* null mutants displayed significantly higher numbers of transitions between the light and dark compartments, as compared to their WT littermates. Males, but not females, displayed more time in the dark chamber, leading to significantly higher time in the dark in the combined cohort 1. In contrast, no genotype differences were detected on either parameter in either sex in cohort 2. These results indicate a possible minor anxiety-related phenotype in *Shank3B* KO mice, in males but not females, and not replicated between the two cohorts tested.

#### Shank3B knockouts display lower open field exploratory activity

Lower scores on measures of open field locomotion were detected in *Shank3B* KO as compared to WT littermates, across the 30-min test session, in males but not in females in cohort 1, and in both sexes in cohort 2. As shown in Additional file 1: Table S4, horizontal activity was lower in male *Shank3B* KO in both cohorts 1 and 2, while female *Shank3B* KO were lower in only cohort 2, with combined male + female scores significantly lower in both cohorts. The pattern of sex and cohort differences was seen on vertical activity and total distance traveled. Center time was lower in male *Shank3B* KO in both cohorts, while females did not differ between genotypes on either cohort, and combined male + female scores were significantly lower on both cohorts. These findings support an interpretation of mild to moderate hypoactivity in *Shank3B* KO, primarily in the males, and consistent with the home cage actigraphy results reported above (*Shank3B* KO mice are hypoactive). These data further highlight the limitations of using open field center time to draw conclusions about anxiety-related behaviors, since horizontal, vertical, and total distance traveled directly affect the center time parameter.

#### Shank3B KO display mostly normal novel object recognition

Cognitive abilities on the novel object recognition task were normal overall in cohort 1 *Shank3B* KO males. As shown in Additional file 1: Table S5, WT and *Shank3B* KO males displayed normal novel object recognition, spending more time sniffing the novel mouse than sniffing the novel object. *Shank3B* KO females showed a trend toward normal novel object recognition, which did not reach statistical significance (*p* = 0.09). During the earlier familiarization session, *Shank3B* KO males showed less total exploration of both identical objects, while female *Shank3B* KO and WT showed similar levels of exploration of both identical objects. Results from this control measure suggest that lower general exploration may account for the apparent novel object recognition deficit in male *Shank3B* KO mice, consistent with their lower open field exploration scores. These findings highlight the value of reporting novel object recognition results as number of seconds spent sniffing each object, rather than as an index, to reveal potential artifacts that could limit the interpretation of a cognitive deficit.

#### Acoustic startle and prepulse inhibition of acoustic startle

Acoustic startle differed between genotypes at higher decibel levels in both cohorts. As shown in Additional file 1: Table S6, startle amplitude was lower in *Shank3B* than in WT at decibel levels of 90, 100, 110, and 120 dB in males for both cohorts 1 and 2, and at 100 and 120 dB in females in cohort 1.

Prepulse inhibition of acoustic startle did not differ between genotypes across prepulse levels from 74 to 92 dB, preceding a startle stimulus of 110 dB, in males, females, or combined males + females, in either cohort 1 or 2.

#### Shank3B KO mice display high levels of repetitive self-grooming, no spontaneous motor stereotypies, and lower marble burying than WT

Observations of behaviors in a clean empty cage did not detect any motor stereotypies in any genotype, sex, or cohort.

**Fig. 5:**
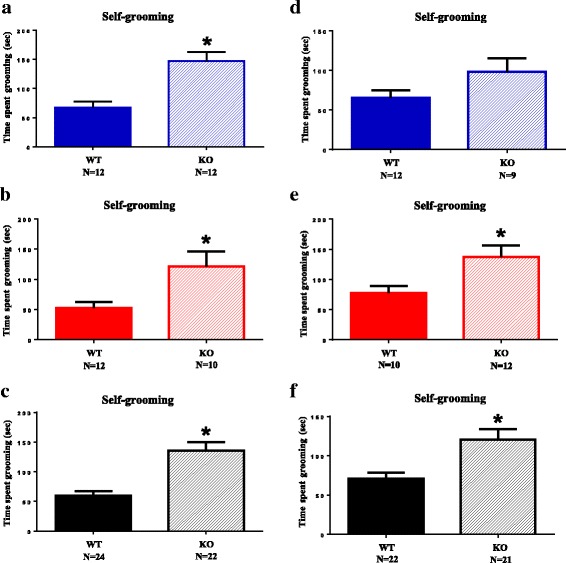
Self-grooming. *Shank3B* null mutant mice (*KO*) spent more time in self-grooming during a 10-min session in a clean empty cage, as compared to wildtype littermates (*WT*), replicated in two independent cohorts. **a** Males, cohort 1, *p* < .001. **b** Females, cohort 1, *p < .02. **c** Combined males and females, cohort 1, *p < .001. **d** Males, cohort 2, *p* = .09. **e** Females, cohort 2, *p < .05, **f** Combined males and females, cohort 2, *p < .01

Repetitive self-grooming was significantly higher in *Shank3B* KO as compared to WT littermates. As shown in Fig. 5 and Additional file 1: Table S7, cumulative time spent grooming during the 10-min test session was significantly higher in *Shank3B* KO males, females, and combined males + females in cohort 1, and in *Shank3B* KO females and combined males + females in cohorts 2, with a trend for higher grooming in cohort 2 males, *p* = .09. These results replicate and extend the original report [16], indicating a robust, replicable elevation in a repetitive behavior as a consequence of the *Shank3B* mutation.

Marble burying scores indicated lower number of marbles buried by both male and female *Shank3B* mice as compared to WT controls in cohort 2, as shown in Additional file 1: Table S7. This finding is inconsistent with an interpretation of more repetitive behaviors in *Shank3B* KO mice, and/or raises questions about whether marble burying represents a repetitive behavior. As the marble burying test was added to the battery only for cohort 2, this finding will require replication in a third cohort.

#### Shank3B KO mice display mostly normal sociability on three-chambered social approach

Sociability in the three-chambered social approach test, defined as more time spent in the chamber with the novel same-sex 129/ImJ target mouse than in the chamber with the novel inverted wire cup object, and more time spent sniffing the novel mouse than sniffing the novel object, was detected in WT male and female mice in both cohorts. As shown in Additional file 1: Figure S1 and Table S8, cohort 1 *Shank3B* KO males displayed significant sociability on chamber time and on sniff time, replicating findings in Peca et al. 2011 [16], but cohort 2 *Shank3B* KO males failed to show significant sociability on chamber time or on sniff time. Female *Shank3B* KO displayed significant sociability on both parameters in both cohorts. This phenotype could be described as a weak sociability deficit only in males, on the three-chambered social approach assay, with the interpretation that *Shank3B* KO null mutants display insufficient phenotypic robustness and replicability on three-chambered social approach for use of this assay in pharmacological intervention studies. It is further interesting to note that the absence of sociability in *Shank3B* KO males in cohort 2 indicates that the apparent deficit on three-chambered social approach is variable across cohorts. This result underscores the importance of testing more than one cohort of mutant mice before drawing firm conclusions about a phenotype caused by the mutation.

#### Shank3B KO mice display normal sensory responses on olfactory habituation/dishabituation and normal hot plate

Evaluation of time spent sniffing a series of non-social and social odors showed that both WT and *Shank3B* KO males and females of both cohorts displayed similar responses to non-social and social odors. As shown in Additional file 1: Table S9, habituation to three water swabs, dishabituation to the first banana swab, habituation to the second and third banana swabs, dishabituation to the first vanilla swab, habituation to the second and third vanilla swabs, dishabituation to the first social cage odor swab, habituation to the second and third swabs from the same first social cage, dishabituation to the first swab from another social cage, habituation to the second and third swabs from the second social cage, were identical between genotypes, for both males and females, in cohort 1. Pain sensitivity on a standard hot plate test was identical between genotypes, for both males and females, in cohort 1. Due to the labor-intensive scoring of the olfactory assay, and the absence of significant genotype differences on the olfactory and nociception tests, these were not repeated in cohort 2.

#### Shank3B KO males display deficits on some parameters of reciprocal social interactions with an unfamiliar WT female in estrus

**Fig. 6:**
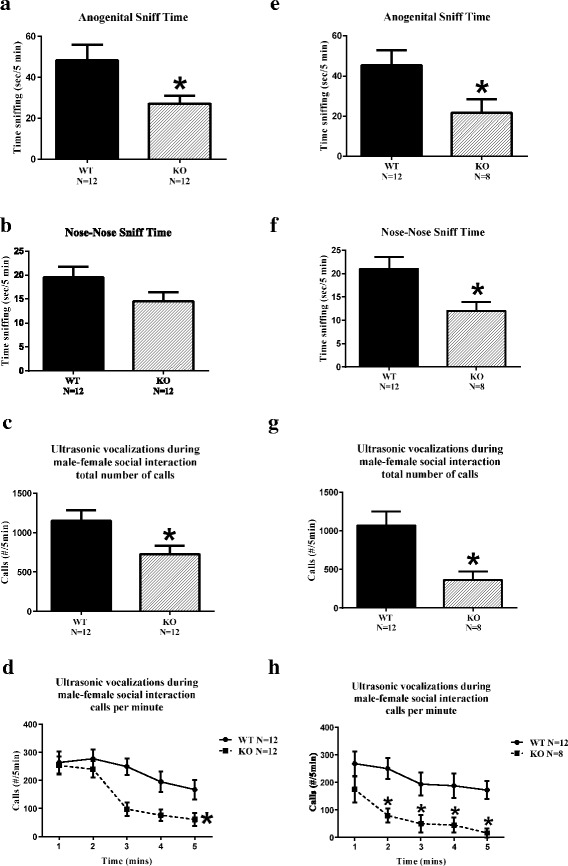
Social interactions. *Shank3B* male null mutant mice (KO) interacting with an estrus WT female displayed deficits on some parameters of reciprocal social interactions, as compared to wildtype littermate males (*WT*), replicated in two independent cohorts. Time spent in nose-to-anogenital sniffing over the 5-min test session was less in *Shank3B* KO than WT (**a** cohort 1, *p < .05; **e** cohort 2, *p < .05). Time spent in nose-to-nose sniffing was not significant in cohort 1 (**b**), but reached significance in cohort 2 (*p < .01). Ultrasonic vocalizations emitted during the 5-min male-female interaction sessions were lower in KO than WT in both cohorts on total number of calls (**c** cohort 1, *p < .02; **g** cohort 2, *p < .02) and on calls per minute (**d** cohort 1, *p < .01; **h** cohort 2, *p < .02)

As shown in Fig. 6 and Additional file 1: Table S10, WT adult males displayed high scores on parameters of sniffing, following, and physical contact with an estrous female, and high levels of ultrasonic vocalizations in the presence of the female. *Shank3B* KO adult males displayed lower scores on some parameters of freely moving reciprocal social interactions with a female. In cohort 1, parameters on which *Shank3B* KO males were significantly lower than WT males included time spent in nose-to-anogenital sniffing, total sniffing time, total number of ultrasonic vocalizations over the 5-min test session, and number of ultrasonic vocalizations analyzed by 1-min time bins. In cohort 2, *Shank3B* KO males showed less time spent in nose-to-anogenital sniffing, less time spent in nose-to-nose sniffing, fewer bouts of nose-to-nose sniffing, emitted significantly fewer total ultrasonic vocalizations over the 5-min test session, and fewer ultrasonic vocalizations analyzed by 1-min time bins. A separate measure of exploration time during the 5-min male-female interaction session, scored from videos in cohort 2 only, did not detect a genotype difference in either time spent in exploration or in number of exploration bouts. While consistency across the two cohorts was not precise for each parameter of male-female social interaction, generally good agreement is seen in most cases between the two cohorts, indicating a relatively high level of robustness for *Shank3B* KO male deficits on this social assay.

#### Shank3B KO mice display normal contextual and cued fear conditioned learning and memory

*Shank3B* KO showed normal freezing scores to context and to the auditory cue, 24 and 48 h after the fear conditioning training session, respectively. As shown in Additional file 1: Table S11, no genotype differences were detected during the contextual test conducted 1 day after training nor during the cued test conducted 2 days after training, for either males or females in cohort 1. *Shank3B* KO showed significantly more freezing immediately following footshock on the training day, as compared to WT, in the males only. This difference usually represents a greater sensory reactivity to the footshock. However, the nociceptive response was normal on the hot plate test, and reactivity to a loud tone was less on the acoustic startle test.

#### Shank3B KO mice display some deficits on Morris water maze acquisition learning and memory

Spatial learning on the Morris water maze detected some genotype differences overall, repeated measures ANOVA F(4,51, *p* < .05). As shown in Additional file 1: Table S12, cohort 1 male *Shank3B* KO differed from male WT on training day 6 and female *Shank3B* KO differed from female WT on training day 7, on latency to reach the hidden platform. Swim speed and distance swum were generally similar between genotypes. Selective search of the trained quadrant of the pool was significant for both WT and *Shank3B* KO males, indicating normal hippocampal-dependent learning using distal spatial cues. Selective quadrant search during the probe trial was significant in WT females but not significant in *Shank3B* KO females, and not significant for the combination of male + female, indicating that learning the location of the hidden platform using distal spatial cues was incomplete. Apparent water maze deficits in females only will require replication in a second cohort.

## Discussion

Reliable, objective, and quantitative biological markers that translate across species remain an unmet need in ASD therapeutic drug development. Our report is the first comparison of such markers, both electrophysiological and behavioral, in separate cohorts of a genetic ASD mouse strain. Encouragingly, we find that most metrics that define the *Shank3B* KO are replicated in two mutant mouse cohorts.

Quantitative abnormalities in neurophysiology have been reported in subsets of individuals with ASD, as well as in some animal models of ASD, suggesting that EEG signatures may be promising markers for ASD patient stratification and treatment response monitoring [56–59]. For example, enhanced EEG gamma oscillatory power has been reported across a number of genetic mouse ASD models, consistent with resting state EEG findings in individuals with neurodevelopmental disorders [31, 60, 61]. Such examples include increased gamma power in both *Mecp2* and *Pten* mutant mouse models [62, 63]. In the present study, we found that male *Shank3B* KO mice have a decreased susceptibility to PTZ induction of all forms of seizures and an enhancement of power in the EEG gamma oscillatory band preceding seizure induction. In line with our objective to identify reliable biomarkers, this finding was replicated in two independent *Shank3B* KO cohorts. This phenotype is consistent with the initial Feng Laboratory assessment that stress-induced seizures were rarely observed in the *Shank3B* KO mouse and consistently spontaneous seizures were never observed [16].

EEG abnormalities, including seizures and subclinical epileptiform activity, are prevalent in both PMS and idiopathic ASD, consistent with the hypothesis that excitatory-inhibitory balance is widely disrupted in ASD [30–32]. Importantly, EEG can be similarly measured in both rodents and humans, and thus EEG phenotypes have realistic translational relevance [33]. Our EEG findings contrast to some degree with the clinical picture of patients with *SHANK3* mutations, as some patients with loss of function *SHANK3* mutations have epilepsy and 67% have some EEG abnormality [31]. Yet, patients with *SHANK3* mutations exhibit considerable heterogeneity in seizure frequency, and a subset may, potentially, resemble the mouse phenotype described above. A prospective natural history study of EEG and epilepsy in individuals with *SHANK3* mutations will help determine to what degree the EEG abnormalities we observed in this mouse model relate to the clinical population.

The electrophysiological phenotype uncovered in the present study suggests the enhancement of inhibitory tone in *Shank3B* KO mice. Notably, in contrast to findings in our null mutant mouse population, EEG of mutant mice with *SHANK3* duplication showed hyperexcitability discharges and electrographic seizures as compared to WT littermates [64]. Thus, *SHANK3* levels—both too little and too much—appear to affect inhibitory neurotransmission. At a cellular level, EEG gamma-oscillations are likely generated by networks of parvalbumin (PV) cells, the most abundant subtype of GABAergic interneurons that contributes to perisomatic inhibition of glutamatergic principal cells [65, 66]. Thus, EEG power in the gamma frequency band reflects the integrity of PV circuitry [67, 68]. PV cell loss in the hippocampus and neocortex is associated with progression to spontaneous seizures after status epilepticus as well as other epileptogenic injuries [69]. Inborn PV cell deficiency also increases seizure susceptibility [70]. Recent meta-analysis indicates that the function and absolute number of PV cells are deficient in mouse ASD models [71], and from this one can hypothesize that gamma EEG power would be lower in the mutant mouse strains. Yet, in our *Shank3B* KO cohorts, we found enhanced gamma EEG power and reduced seizure susceptibility.

The above findings are consistent with mitigation for seizure risk by enhanced cortical inhibition as reflected in the EEG gamma band. Alternatively, the reduced seizure susceptibility in the *Shank3B* strain may reflect reduced excitatory tone, rather than enhanced inhibitory tone. The high power in the gamma frequency band of the *Shank3B* KO mouse model is also a plausible readout of high PV cell network activity responsible for heightened seizure threshold. Perhaps, this reflects a compensatory over-activation of the PV inhibitory system in the setting of increased seizure vulnerability in ASD. Yet, independent of the specific mechanism, our data raise prospects for some patients with *SHANK3* mutations to also have a mild or absent epilepsy phenotype. Further, while beyond the scope of this report, such finding of seizure protection in a *Shank3B* KO mutant indicates a potential to manipulate the *SHANK3* gene or protein product as a means to stop or prevent epileptic seizures.

ASD is diagnosed and defined by behavioral symptoms in the domains of (a) social interaction and communication and (b) stereotyped, repetitive behaviors with restricted interests. However, considerable heterogeneity characterizes the broad range of diagnostic and associated symptoms across individuals [56, 72]. Similarly, mouse models of ASD with mutations in risk genes for ASD vary considerably in their expression of social and repetitive abnormalities, and in phenotypes relevant to the cognitive, anxiety, sensory, and motor abnormalities associated with many cases of ASD [50]. One issue in the current literature is the extent to which variability in observed behavioral phenotypes in a mouse model of ASD may arise from procedural or environmental issues, such as differences in housing conditions or animal handling practices. Other issues include the use of non-standard methods and incorrect interpretations of behavioral results. In most cases, the behavioral phenotypes reported in the first publication of a mutant line of mice have not yet been repeated in follow-up cohorts, either by the same or other laboratories. In cases in which follow-up studies were conducted, findings were replicated in many cases [43, 44, 49, 73, 74]. However, findings have not replicated in other cases, e.g. [45], and anecdotal reports suggest that failures to replicate have not yet been published. Similar issues may arise in the future as more laboratories engage in assaying physiological parameters in rodent models of ASD.

To improve the utility of genetic mouse models of ASD pathogenesis, as a part of our collaborative Autism Speaks Preclinical Autism Consortium for Therapeutics (PACT), we are investigating behavioral and translational physiological phenotypes in replication cohorts of mice with published mutations in risk genes for ASD and related conditions. To this end, we conducted in-depth phenotyping of the *Shank3B* null mutant mouse model originally generated by Guoping Feng and coworkers at Duke University [16]. These mice harbor the *Shank3* mutation in the PDZ domain and were reported to display remarkably high levels of repetitive self-grooming and social deficits [16]. Of note, similar results were reported by Craig Powell and coworkers using a different genetic manipulation of the *Shank3* gene [19]. Our larger goal for the present report and for related PACT studies is to identify behavioral phenotypes that replicate consistently in independent cohorts of mice within and between laboratories, in order to strengthen the use of preclinical mouse models of ASD, (a) for understanding the mechanistic underpinnings of ASD-relevant phenotypes, and (b) for preclinical translation in evaluating the therapeutic potential of novel treatments.

In the present studies, we employed a highly-standardized set of behavioral testing methods to identically evaluate two separately bred cohorts of *Shank3B* KO and their WT littermates, both males and females, in a fixed sequence of behavioral assays. Overall strategy, techniques and methods, testing batteries, and test sequence were developed as a component of PACT, in close collaboration among Drs. Crawley and Sahin and senior leadership of the Autism Speaks scientific research team. In addition to translational neurophysiological markers, the PACT preclinical platform was designed to evaluate behaviors relevant to the diagnostic and associated symptoms of autism, including social, repetitive, cognitive, anxiety-related, sensory, and motor traits, in multiple lines of genetic mouse models, across two independent cohorts and at least two corroborative tests within each behavioral domain. Two cohorts, each designed to yield Ns of 10 per genotype and sex, were independently bred and tested, to include a comparison of sex as a biological variable, for optimal experimental design in testing potential therapeutics.

Similar results were obtained between the two cohorts on most, but not all, of the ASD-relevant social and repetitive behavior assays conducted. The strongest ASD-relevant phenotype in *Shank3B* KO mice was repetitive self-grooming, as originally reported [16]. Grooming scores were almost twice as high in *Shank3B* KO as compared to their WT littermates. Time spent self-grooming was significant for both male and female KO and their combined scores in cohort 1, and for females and the combination of males and females in cohort 2. These findings support the interpretation that repetitive self-grooming is a robust and reproducible feature of *Shank3B* KO mice. Repetitive behaviors are a common manifestation in individuals with Phelan-McDermid Syndrome [75], seen in over half the patients. Thus, *Shank3B* KO mice offer a robust animal model for future studies to develop treatments for repetitive behaviors.

In the social domain, reciprocal social interactions in same-sex dyads at juvenile age 22–28 days old were not significantly different between genotypes, in both cohorts 1 and 2. Three-chambered social approach, an assay developed by our group in 2004 [36, 37], revealed normal sociability in *Shank3B* KO males in cohort 1 and in *Shank3B* KO females in cohorts 1 and 2, but absence of sociability in *Shank3B* KO males in cohort 2. The more sensitive, nuanced assay of reciprocal social interactions between freely moving subject males and WT estrous females showed strong genotype differences on some parameters in both cohorts, including ultrasonic vocalizations emitted. Other parameters of reciprocal social interactions did not differ between genotypes. For behavioral assays with multiple outcome parameters, interpretations may best be based on the preponderance of significant genotype differences across several related parameters. The preponderance of significantly less sniffing and vocalizing during male-female reciprocal social interactions in two cohorts, but some normal scores on three-chambered social approach, emphasizes the need to conduct more than one behavioral assay and to select the strongest outcome measures of the mutation to employ in therapeutic discovery.

Results that differ between cohorts are the most difficult to interpret. For example, if interpretations had been based on three-chambered sociability results from only the second cohort, we would have concluded that sociability was impaired in *Shank3B* KO males but not females, and focused the discussion on an interesting sexual dimorphism relevant to the higher prevalence of ASD in boys than girls. However, since sexual dimorphism appeared in only one of two cohorts, interpretations must be more cautious. Further caution would be extended to the use of our simpler automated three-chambered social approach task as the sole social assay for preclinical therapeutic discovery in the *Shank3B* KO line of mice. Social assays with higher sensitivity, such as male-female interactions, and new assays with greater face validity to the types of social abnormalities that characterize autism, may improve translational success.

Open field exploration was reduced in *Shank3B* KO in males in cohort 1, and in both sexes in cohort 2, representing a relatively strong and reproducible phenotype. Lower scores on several open field parameters in *Shank3B* KO mice, detected in the Crawley lab, is consistent with the *Shank3B* KO displaying significantly less activity in circadian home cages in the Sahin lab. While reduced activity introduced a potential confound in interpreting light↔dark anxiety-related behavior and fear conditioning, as described below, the magnitude of locomotor differences did not appear to be large enough to impact performance on other assays, as measured by internal activity parameters during performance in other tests including three-chambered social approach and novel object recognition assays.

Anxiety-related tests produced variable results. On the elevated plus-maze, no genotype differences were detected in either cohort on either of the two anxiety-related parameters, percent open arm time and number of open arm entries. However, total number of entries, the control measure for locomotion during elevated plus-maze testing, showed less exploratory activity in *Shank3B* KO males, but not in *Shank3B* KO females, and in Cohort 1 only, suggesting overall normal performance on this anxiety-related test. Light↔dark transitions were significantly lower in both male and female *Shank3B* KO in Cohort 1, and time in the dark was higher in Cohort 1 males. However, in Cohort 2, all parameters of light↔dark anxiety-like behavior and locomotion showed no genotype differences in either sex. An anxiety-like phenotype in one cohort but not in the other cohort is best interpreted as a minor finding, of insufficient replicability to use in a treatment study. It is interesting to note that comparison by sex revealed behaviors in which similar phenotypes were detected for both males and females, and behaviors in which genotype differences were significant in only one sex, either male or female in the anxiety-related domain and in other behavioral assays. These results highlight the usefulness of displaying results by sex as well as a combined total, when confirming replicability.

Marble burying was lower in *Shank3B* KO, rather than higher as predicted from the assumption that marble burying is a repetitive behavior. Both male and female *Shank3B* KO buried fewer marbles as compared to same-sex WT. This unexpected result adds to an existing question in the behavioral neuroscience field about whether marble burying represents a repetitive behavior, an anxiety-related behavior, an artifact of digging in deep litter, or something else [76]. It seems possible that a competing behavior such as self-grooming was responsible for less marble burying in *Shank3B* KO. However, as marble burying was not conducted in cohort 1, but added as a corroborative task in cohort 2, findings will require replication in a third cohort.

Sensory abnormalities appeared on some assays. Reduced acoustic startle to loud decibel level white noise stimuli indicate somewhat reduced hearing or reduced perception of startle stimuli. In contrast, no genotype differences in prepulse inhibition were detected in either sex in either cohort. Olfactory abilities in the olfactory habituation/dishabituation assay were normal for *Shank3B* KO females but showed some impairments in *Shank3B* KO males, a finding that will require replication in another cohort. Hot plate response latencies did not differ between genotypes, indicating normal nociception on this gross measure of pain sensitivity.

Cognitive abilities appeared to be mostly intact in *Shank3B* KO mice on novel object recognition, contextual and cued fear conditioning, and Morris water maze acquisition. Female *Shank3B* KO displayed borderline deficits on novel object recognition and failed the water maze probe trial. These variable findings justify future studies to repeat the cognitive tests in a future cohort of *Shank3B* KO and WT mice. While intellectual impairments, anxiety, hyperactivity, and unusual responses to sensory stimuli are associated symptoms rather than diagnostic symptoms of ASD, and therefore were not the primary focus of our PACT experimental design, strong phenotypes in an associated symptom domain could provide valuable additions to a mouse model of ASD when evaluating potential therapeutics.

## Conclusions

Replicable and quantitative biomarkers of ASD pathophysiology are essential to the utility and value of mouse models of ASD in the discovery of efficacious therapies. The *Shank3B* KO genetic mouse model of ASD, due to its molecular and biochemical convergence with other ASD-related risk genes, as well as the findings of the present study and cumulative literature on *SHANK3* models, is a strong and a well-validated model that appears to be a promising tool to screen novel pharmacological therapeutics. Our studies are the first in vivo physiology analysis of *Shank3B* null mutant mice, to our knowledge. This model displays robust, replicated phenotypes on measures of baseline EEG and PTZ-induced seizure susceptibility. Importantly, EEG is a quantitative readout of neural activity that can be measured in mice and humans and therefore has the potential to serve as a translational biomarker for patient stratification or treatment efficacy. While EEG has not yet been reported as a sensitive marker of pharmacological interventions, it was shown be sensitive to behavioral intervention in children with ASD [77]. It is important to note that the present studies compared *Shank3B* null mutants to wildtype littermate controls, whereas the human mutation is generally heterozygous. Future investigation of *Shank3B* heterozygote mice may yield further insights. To further facilitate the translation of successful preclinical pharmacological treatment studies to successful clinical trials, behavioral assays were conducted in parallel with characterization of EEG parameters in replication cohorts of *Shank3B* null mutant mice. Our findings reveal that this model has robust, replicated phenotypes on measures of baseline EEG and PTZ-induced seizure susceptibility. Decreased seizure susceptibility and increased high frequency gamma oscillatory power are indicative of increased inhibitory tone in the null mutants. In addition, in two independent cohorts of *Shank3B* KO mice and their WT littermates, we replicated high levels of repetitive self-grooming and impairments in parameters of male-female reciprocal social interactions. These behavioral results corroborate and expand on elements of the initial characterization by the Feng Laboratory [16].

Taken together, our findings support the use of *Shank3B* null mutant mice as a stable and appropriate mouse model of ASD for therapeutic discovery. Both the robust and replicable behavioral phenotypes and the translational and quantitative electrophysiological phenotypes identified in this study should empower the ASD research field to utilize this model for the discovery and characterization of potential new treatments.

## Additional files

Additional file 1: Tables S1. Sequence of longitudinal behavioral testing across developmental ages. Order of testing was conducted identically in two independently bred and tested cohorts of WT and *Shank3B* null mutant mice. **Table S2.** Juvenile reciprocal social interactions were normal in *Shank3B* mice. **Table S3.** Anxiety-related behaviors in *Shank3B* and WT mice. **Table S4.** Reduced open field activity in *Shank3B* mice. **Table S5.** Normal novel object recognition in *Shank3B* mice. **Table S6.** Acoustic startle and prepulse inhibition in *Shank3B* mice. **Table S7.** High levels of repetitive self-grooming in *Shank3B* mice. **Figure S1.** Three-chambered social approach in *Shank3B* mice. **Table S8.** Three-chambered social approach in *Shank3B* mice. **Table S9.** Olfactory habituation/dishabituation and hot plate nociception sensory phenotypes. **Table S10.** Adult male-female reciprocal social interactions. **Table S11.** Contextual and cued fear conditioning. **Table S12.** Morris water maze acquisition and probe trial. (PDF 895 kb)

Additional file 2: Figure S2. EEG low frequency power spectral analysis. (PDF 59 kb)

## Abbreviations

ANOVA: Analysis of variance
ASD: Autism spectrum disorder
EEG: Electroencephalography
FFT: Fast fourier transform
GTCS: Generalized tonic-clonic seizure
i.p.: Intraperitoneal
KO: Knockout
PACT: Preclinical Autism Consortium for Therapeutics
PC: Personal computer
PMS: Phelan-McDermid Syndrome
PTZ: Pentylenetetrazol
WT: Wildtype
