# Supplementary material for: Replicable in vivo physiological and behavioral phenotypes of the Shank3B null mutant mouse model of autism
Source: Mol Autism. 2017 Jun 15;8:26. doi: 10.1186/s13229-017-0142-z (PMC5472997; doi:10.1186/s13229-017-0142-z)
Supplement: Supplementary file 1 — Sequence of longitudinal behavioral testing across developmental ages. Order of testing was conducted identically in two independently bred and tested cohorts of WT and Shank3B null mutant mice. Table S2. Juvenile reciprocal social interactions were normal in Shank3B mice. Table S3. Anxiety-related behaviors in Shank3B and WT mice. Table S4. Reduced open field activity in Shank3B mice. Table S5. Normal novel object recognition in Shank3B mice. Table S6. Acoustic startle and prepulse inhibition in Shank3B mice. Table S7. High levels of repetitive self-grooming in Shank3B mice. Figure S1. Three-chambered social approach in Shank3B mice. Table S8. Three-chambered social approach in Shank3B mice. Table S9. Olfactory habituation/dishabituation and hot plate nociception sensory phenotypes. Table S10. Adult male-female reciprocal social interactions. Table S11. Contextual and cued fear conditioning. Table S12. Morris water maze acquisition and probe trial. (PDF 895 kb) [file 13229_2017_142_MOESM1_ESM.pdf]

Supplementary Materials

Table S1
Sequence of longitudinal behavioral testing across developmental ages. Order of testing was conducted identically in two independently bred and tested cohorts of WT and *Shank3B* null mutant mice

Postnatal day	Behavioral assay	Domain
**24-26**	**Juvenile reciprocal interactions with ultrasonic vocalizations**	Social
**30-34**	**Elevated plus-maze and light↔dark transitions**	Anxiety
**36-38**	**Open field exploratory locomotion**	Activity
**40-42**	**Novel object recognition**	Cognitive
**44-46**	**Acoustic startle threshold and prepulse inhibition of startle**	Sensory
**48-50**	**Spontaneous motor stereotypies and repetitive behaviors**	Repetitive
**52-56**	**Social approach 3-chambered in young adults**	Social
**58-62**	**Olfactory habituation/dishabituation**	Sensory
**64-65**	**Hot plate**	Sensory
**67-74**	**Male-female adult social interaction with ultrasonic vocalizations**	Social
**76-79**	**Contextual and cued fear conditioning**	Cognitive
**81-88**	**Morris water maze acquisition and reversal**	Cognitive

Table S2

Juvenile reciprocal social interactions were normal in *Shank3B* mice

	COHORT 1	COHORT 2
a) Nose-to-nose sniffing (cumulative time in seconds)		
Males	F(1,22)=.601, NS	F(1,6)*=.065, NS
Females	F(1,20)=.824, NS	F(1,7)=.310, NS
Combined	F(1,44)=.142, NS	F(1,15)=.387, NS
b) Nose-to-nose sniffing (number of bouts)		
Males	F(1,22)=.138, NS	F(1,6)=.000, NS
Females	F(1,20)=.867, NS	F(1,7)=.454, NS
Combined	F(1,44)=.083, NS	F(1,15)=.304, NS
c) Nose-to-anogenital sniffing (sec)		
Males	F(1,22)=.039, NS	F(1,6)=.686, NS
Females	F(1,20)=.532, NS	F(1,7)=.362, NS
Combined	F(1,44)=.357, NS	F(1,15)=.012, NS
d) Nose-to-anogenital sniffing (bouts)		
Males	F(1,22)=1.43, NS	F(1,6)=.019, NS
Females	F(1,20)=.013, NS	F(1,7)=1.24, NS
Combined	F(1,44)=.758, NS	F(1,15)=.736, NS
e) Following (sec)		
Males	F(1,22)=.080, NS	F(1,6)=.602, NS
Females	F(1,20)=3.44, NS	F(1,7)=.144, NS
Combined	F(1,44)=2.50, NS	F(1,15)=.121, NS
f) Following (bouts)		
Males	F(1,22)=.006, NS	F(1,6)=.259, NS
Females	F(1,20)=5.04, p<.05	F(1,7)=.138, NS
Combined	F(1,44)=1.05, NS	F(1,15)=.335, NS
g) Front approach		
Males	F(1,22)=.015, NS	n.d.**
Females	F(1,20)=1.06, NS	n.d.

Table S2. Juvenile reciprocal interactions between a 24-26 day old *Shank3B* null mutant (KO) subject mouse and a sex- and age-matched wildtype littermate (WT) mouse for 10 minutes in a Noldus Phenotyper 3000. Videos were recorded and subsequently scored on representative parameters, using Noldus Ethovision software, by a trained observer uninformed of genotype. Statistical values represent genotype comparisons for each parameter. No genotype differences were detected on parameters of nose-to-nose sniffing, nose-to-anogenital sniffing, following, as measured either in time spent or number of bouts of each parameter, with the exception of lower number of following bouts in female KO. Front approach did not differ between genotypes in Cohort 1; **front approach was not scored in Cohort 2.

Table S3
Anxiety-related behaviors in *Shank3B* and WT mice

Elevated plus-maze

	COHORT 1	COHORT 2
a) % Open arm time		
Males	F(1,22)=.729, NS	F(1,19)=1.60, NS
Females	F(1,20)=.001, NS	F(1,20)=1.78, NS
Combined	F(1,44)=.285, NS	F(1,41)=3.70, NS
b) Open arm entries		
Males	F(1,22)=.858, NS	F(1,19)=2.15, NS
Females	F(1,20)=.816, NS	F(1,20)=6.89, p<.02
Combined	F(1,44)=.884, NS	F(1,41)=8.94, p<.01
c) Total entries		
Males	F(1,22)=6.18, p<.05	F(1,19)=0.548, NS
Females	F(1,20)=.264, NS	F(1,20)=.032, NS
Combined	F(1,44)=6.95, p<.02	F(1,41)=.585, NS

Light↔dark transitions

	COHORT 1	COHORT 2
a) # Transitions		
Males	F(1,22)=14.4, p<.01	F(1,11)=1.34, NS
Females	F(1,20)=9.62, p<.01	F(1,11)=4.23, NS
Combined	F(1,44)=15.9, p<.01	F(1,24)=3.25, NS
b) Time in dark side		
Males	F(1,22)=7.89, p<.05	F(1,11)=1.39, NS
Females	F(1,20)=3.41, NS	F(1,11)=.023, NS
Combined	F(1,44)=10.6, p<.01	F(1,24)=.732, NS

Table S3. Elevated plus-maze scores showed no genotype differences on percent time spent in the open arms in Cohorts 1 and 2, and no difference in number of entries into the open arms in Cohort 1 and in males in Cohort 2. Females in Cohort 2 and combined male + female scores for Cohort 2 showed fewer entries into the open arms. Total entries were lower in males and in combined males and females in Cohort 1 only. Light↔dark transitions showed anxiety-like scores in Cohort 1 on number of transitions between the light and dark compartments in both males and females of Cohort 1 but not in Cohort 2. Time spent in the dark compartment was greater in males and in the score for combined males and females, in Cohort 1 only. These inconsistent data indicate considerably variability between the two cohorts, and possible sex differences, on two anxiety-related tasks. Reduced exploratory activity in the

open field test (Table S4), taken together with reduced number of entries in Cohort 1 males, limits the interpretation of anxiety-like phenotypes in *Shank3B* mice.

Table S4
Reduced open field activity in *Shank3B* mice

	COHORT 1	COHORT 2
a) Horizontal activity		
Males	F(1,22)=53.86, p<.001	F(1,19)=5.11, p<.05
Females	F(1,20)=.747, NS	F(1,20)=18.6, p<.001
Combined	F(1,44)=18.9, p<.001	F(1,41)=18.7, p<.001
b) Vertical activity		
Males	F(1,22)=32.7, p<001	F(1,19)=1.94, p<.05
Females	F(1,20)=2.80, NS	F(1,20)=5.41, p<.05
Combined	F(1,44)=21.7, p<.001	F(1,41)=7.45, p<.01
c) Total distance traveled		
Males	F(1,22)=28.9, p<.001	F(1,19)=4.92, p<.05
Females	F(1,20)=2.23, NS	F(1,20)=9.35, p<.01
Combined	F(1,44)=14.7, p<.001	F(1,41)=13.2, p<.001
d) Center time		
Males	F(1,22)=8.22, p<.01	F(1,19)=15.0, p<.001
Females	F(1,20)=.053, NS	F(1,20)=1.59, NS
Combined	F(1,44)=5.32, p<.05	F(1,41)=7.14, p<.01

Figure S4. Open field activity in a 30 minute test session in a novel environment showed significantly lower scores in *Shank3B* KO mice as compared to WT on all parameters in males of both Cohort 1 and Cohort 2, and in females on Cohort 2. Moderately reduced general exploration in *Shank3B* mice could have influenced scores on other behavioral assays. However, internal controls for general activity in other assays do not indicate a major contribution of reduced locomotion on performance of other behavioral tasks.

Table S5
Normal novel object recognition in *Shank3B* mice

	COHORT 1
a) Familiarization session	
WT Males	F(1,11)=.001, NS
WT Females	F(1,11)=.051, NS
WT Combined	F(1,23)=.023, NS
Shank3B Males	F(1,11)=.057, NS
Shank3B Females	F(1,9)=.024, NS
Shank3B Combined	F(1,21)=.034, NS
b) Novel object recognition session	
WT Males	F(1,11)=5.19, p<.05
WT Females	F(1,11)=18.5, p<.01
WT Combined	F(1,23)=14.7, p<.001
Shank3B Males	F(1,11)=6.29, p<.05
Shank3B Females	F(1,9)=3.58, p=.091,NS
Shank3B Combined	F(1,21)=8.58, p<.01

Table S5. During the 10 minute familiarization session, all genotypes and sexes explored the two identical objects equally (a, NS), indicating normal exploratory activity. During the 5 minute novel object recognition session (b), conducted 1 hour later, WT males and females, and *Shank3B* males, spent significantly more time sniffing the novel object than the now-familiar object, meet the criterion for normal novel object recognition. Female *Shank3B* showed a trend for normal novel object recognition that did not reach statistical significance (p=.091). Based on mostly normal scores in Cohort 1, and that cognitive deficits are an associated symptom of autism rather than diagnostic, novel object recognition was not tested in Cohort 2.

Table S6
Acoustic startle and prepulse inhibition in *Shank3B* mice

Acoustic startle

Decibel level	COHORT 1	COHORT 2
a) 0		
Males	F(1,22)=3.49, NS	F(1,19)=3.22, NS
Females	F(1,20)=.608, NS	F(1,20)=1.28, NS
Combined	F(1,44)=3.59, NS	F(1,41)=5.27, p<.05
b) 80		
Males	F(1,22)=1.63, NS	F(1,19)=1.48, NS
Females	F(1,20)=2.38, NS	F(1,20)=0.278, NS
Combined	F(1,44)=3.53, NS	F(1,41)=.081, NS
c) 90		
Males	F(1,22)=2.73, NS	F(1,19)=2.94, NS
Females	F(1,20)=2.47, NS	F(1,20)=0.892, NS
Combined	F(1,44)=5.38, p<.05	F(1,41)=4.49, p<.05
d) 100		
Males	F(1,22)=6.01, p<.05	F(1,19)=9.56, p<.01
Females	F(1,20)=5.06, p<.05	F(1,20)=0.836, NS
Combined	F(1,44)=11.6, p<.01	F(1,41)=9.45, p<.01
e) 110		
Males	F(1,22)=6.99, p<.05	F(1,19)=14.2, p<.01
Females	F(1,20)=1.94, NS	F(1,20)=0.119, NS
Combined	F(1,44)=7.41, p<.01	F(1,41)=8.67, p<.01
f) 120		
Males	F(1,22)=14.0, p<.01	F(1,19)=17.2, p<.01
Females	F(1,20)=4.70, p<.05	F(1,20)=1.61, NS
Combined	F(1,44)=13.8,p<.001	F(1,41)=13.6,p<.001

Prepulse inhibition of acoustic startle

	COHORT 1	COHORT 2
Males	F(1,22)= 1.33, NS	F(1,19)= 3.43, NS
Females	F(1,20)= 2.09, NS	F(1,20)= 0.24, NS
Combined prepulse dB	F(1,44)= .042, NS	F(1,41)= 2.08, NS

Table S6. Acoustic startle was reduced in both male and female *Shank3B* males and females as compared to WT littermates at decibel levels of 110 dB, 110 dB, and 120 dB, in both cohorts.

Statistical values represent genotype comparisons for each parameter. These data indicate reduced startle response and/or hearing deficits to the loudest stimuli. Prepulse inhibition did not differ between genotypes when compared across prepulse levels of 0, 74, 78, 82, 85, and 92 dB, preceding a 110 dB acoustic startle stimulus.

Table S7
High levels of repetitive self-grooming in *Shank3B* mice

Self-grooming

	COHORT 1	COHORT 2
Time spent grooming (sec)		
Males	t(1,22)=4.13, p<.005	t(1,19)=1.78, NS
Females	t(1,20)=2.78, p<.05	t(1,20)=2.52, p<.05
Combined	t(1,44)=4.86, p<.001	t(1,41)=3.24, p<.01

Marble burying

	COHORT 2
Number of marbles buried	
Males	F(1,19)= 34.2, p<.001
Females	F(1,20)=41.0, p<.001
Combined	F(1,41)=12.6, p<.005

Table S7. Time spent engaged in grooming during a 10 minute session in a clean empty cage was significantly higher for *Shank3B* KO than WT in Cohort 1 males, Cohort 1 and 2 females, and combined scores for males+females in both cohorts. Statistical values represent genotype comparisons. In contrast, marble burying yielded significant results but in an unpredicted direction. Both male and female KO buried more marbles than WT. Marble burying was not tested in Cohort 1, but was added as a further evaluation of repetitive behavior in Cohort 2.

Figure S1
Three-chambered social approach in *Shank3B* mice

Cohort 1 Males

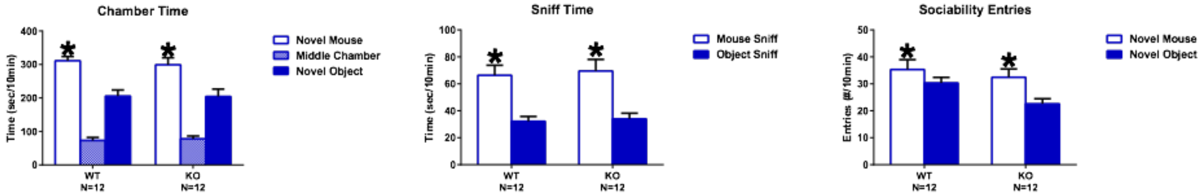

Cohort 1 Females

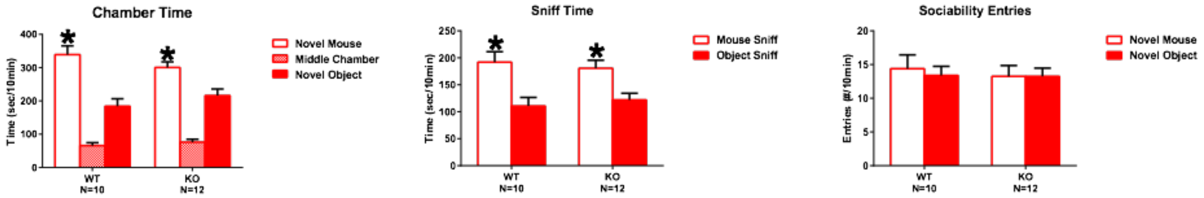

Cohort 1 Combined Males + Females

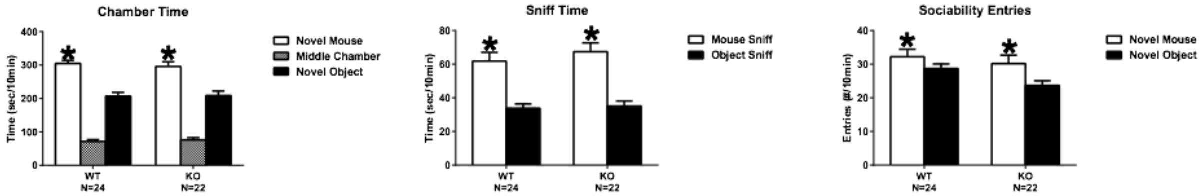

Cohort 2 Males

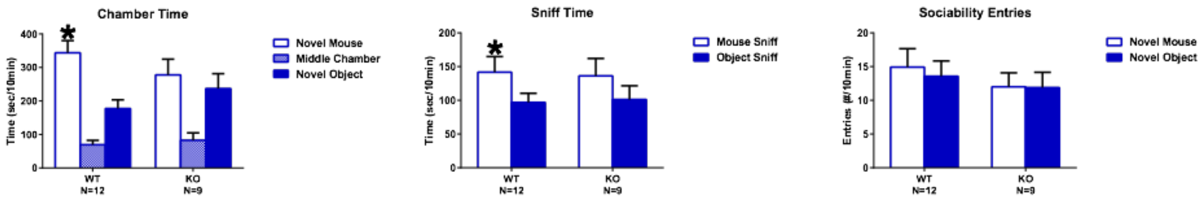

Cohort 2 Females

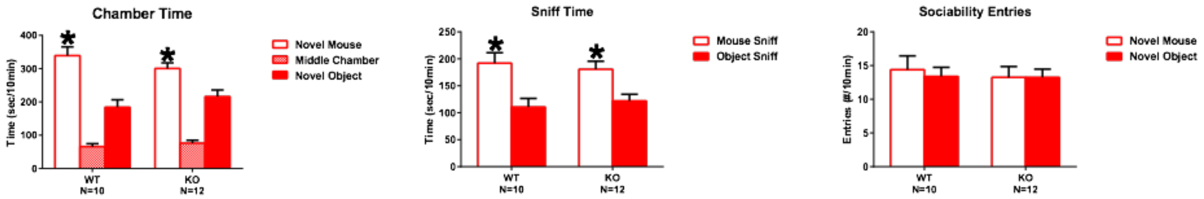

Cohort 2 Combined Males + Females

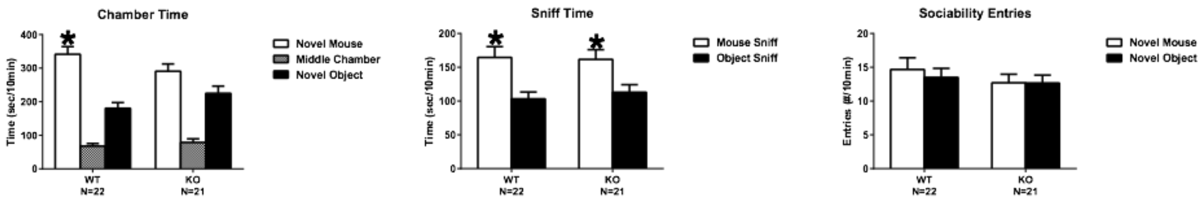

Table S8

Three-chambered social approach in *Shank3B* mice

	COHORT 1	COHORT 2
a) WT Chamber time		
Males	F(1,11)=12.4, p<.01	F(1,11)=8.49, p<.05
Females	F(1,11)=11.5, p<.01	F(1,9)=12.0, p<.01
Combined	F(1,23)=24.9, p<.001	F(1,21)=18.2, p<.001
b) WT Sniff time		
Males	F(1,11)=12.2, p<01	F(1,11)=7.60, p<.05
Females	F(1,11)=8.58, p<.05	F(1,9)=12.8, p<.01
Combined	F(1,23)=20.7, p<.001	F(1,21)=18.1, p<.001
c) WT Chamber Entries		
Males	F(1,22)=5.00, p<.05	F(1,11)=1.13, NS
Females	F(1,20)=.942, NS	F(1,9)=1.97, NS
Combined	F(1,44)=5.38, p<.05	F(1,21)=1.76, NS
d) *Shank3B* Chamber time		
Males	F(1,11)=5.04, p<.05	F(1,8)=.548, NS
Females	F(1,9)=6.12, p<.05	F(1,11)=5.74, p<.05
Combined	F(1,21)=10.8, p<.01	F(1,20)=2.44, NS
d) *Shank3B* Sniff time		
Males	F(1,11)=10.9, p<.001	F(1,8)=3.50, NS
Females	F(1,9)=10.8, p<.01	F(1,11)=7.70, p<.05
Combined	F(1,21)=26.6, p<.001	F(1,20)=9.15, p<.01
c) *Shank3B* Chamber Entries		
Males	F(1,11)=10.9, p<.01	F(1,8)=0.11, NS
Females	F(1,9)=.522, NS	F(1,11)=.000, NS
Combined	F(1,21)=7.67, p<.05	F(1,20)=.002, NS

Figure S1 and Table S8. Three-chambered social approach was normal in most cases in one cohort of male *Shank3B* KO, in both cohorts of female *Shank3B* KO, and in both cohorts of male and female WT, on both the chamber time parameter and the sniffing time parameter. No genotype differences were detected on number of entries, an internal control for locomotion. In this binary yes-or-no automated 10 minute assay, sociability is defined as more time spent in the side chamber containing a novel mouse than in the side chamber containing a novel object, within genotype. A second more sensitive measure of sociability in this assay is defined as more time spent sniffing the novel mouse than sniffing the novel object, within genotype. In our previous experience, neither parameter is sensitive enough to compare the amount of time spent with the novel mouse across genotypes. Derived index measures may be misleading, due to the direct influence of general activity in all three chambers on sociability scores.

Table S9
Olfactory habituation/dishabituation and hot plate nociception sensory phenotypes

Olfactory Habituation/Dishabituation

	COHORT 1
**WT**	
Males	
Habituation to 3 water presentations	F(1,9)=49.1, p<.001
Dishabituation to new banana odor	F(1,9)=4.32, p=.06
Habituation to three banana presentations	F(1,9)=4.13, p=.07
Dishabituation to new vanilla odor	F(1,9)=13.1, p<.01
Habituation to three vanilla presentations	F(1,9)=7.02, p<.05
Dishabituation to new social odor 1	F(1,9)=26.8, p<.001
Habituation to three social odor 1 presentations	F(1,9)=27.4, p<.001
Dishabituation to new social odor 2	F(1,9)=11.5, p<.01
Habituation to three social odor 2 presentations	F1,9)=27.9, p<.001
Females	
Habituation to 3 water presentations	F(1,11)=38.2, p<.001
Dishabituation to new banana odor	F(1,11)=11.0, p<.01
Habituation to three banana presentations	F(1,11)=5.95, p<.05
Dishabituation to new vanilla odor	F(1,11)=10.7, p<.01
Habituation to three vanilla presentations	F(1,11)=14.2, p<..01
Dishabituation to new social odor 1	F(1,11)=40.7, p<.001
Habituation to three social odor 1 presentations	F(1,11)=21.7, p<.001
Dishabituation to new social odor 2	F(1,11)=15.2, p<.01
Habituation to three social odor 2 presentations	F1,11)=28.2, p<.001
Combined males + females	
Habituation to 3 water presentations	F(1,21)=88.3, p<.001
Dishabituation to new banana odor	F(1,21)=11.1, p<.01
Habituation to three banana presentations	F(1,21)=8.56, p<.01
Dishabituation to new vanilla odor	F(1,21)=23.0, p<001
Habituation to three vanilla presentations	F(1,21)=19.1, p<.001
Dishabituation to new social odor 1	F(1,21)=69.8, p<.001
Habituation to three social odor 1 presentations	F(1,21)=49.7, p<.001
Dishabituation to new social odor 2	F(1,21)=27.5, p<001
Habituation to three social odor 2 presentations	F1,21)=55.6, p<.001
Shank3B	
Males	
Habituation to 3 water presentations	F(1,7)=1.85, NS
Dishabituation to new banana odor	F(1,7)=2.01, NS

Habituation to three banana presentations	F(1,7)=4.02, p=.085
Dishabituation to new vanilla odor	F(1,7)=10.0, p<.05
Habituation to three vanilla presentations	F(1,7)=10.9, p<.05
Dishabituation to new social odor 1	F(1,7)=35.5, p<.001
Habituation to three social odor 1 presentations	F(1,7)=37.9, p<.001
Dishabituation to new social odor 2	F(1,7)=3.56, NS
Habituation to three social odor 2 presentations	F(1,7)=2.28, NS
Females	
Habituation to 3 water presentations	F(1,9)=5.37, p<.05
Dishabituation to new banana odor	F(1,9)=19.3, p<.01
Habituation to three banana presentations	F(1,9)=19.2, p<.01
Dishabituation to new vanilla odor	F(1,9)=21.0, p<.01
Habituation to three vanilla presentations	F(1,9)=22.0, p<.01
Dishabituation to new social odor 1	F(1,9)=22.9, p<.001
Habituation to three social odor 1 presentations	F(1,9)=13.9, p<.01
Dishabituation to new social odor 2	F(1,9)=12.1, p<.01
Habituation to three social odor 2 presentations	F(1,9)=8.11, p<.05
Combined males + females	
Habituation to 3 water presentations	F(1,17)=6.46, p<.05
Dishabituation to new banana odor	F(1,17)=8.43, p<.01
Habituation to three banana presentations	F(1,17)=12.6, p<.01
Dishabituation to new vanilla odor	F(1,17)=23.2, p<.001
Habituation to three vanilla presentations	F(1,17)=24.8, p<.001
Dishabituation to new social odor 1	F(1,17)=49.7, p<.001
Habituation to three social odor 1 presentations	F(1,17)=41.6, p<.001
Dishabituation to new social odor 2	F(1,17)=14.2, p<.01
Habituation to three social odor 2 presentations	F(1,17)=9.17, p<.01

Hot plate nociception

	COHORT 1
Latency to reaction (sec)	
Males	F(1,22)= .655, NS
Females	F(1,20)=.003, NS
Combined	F(1,41)=.315, NS

Table S9. Top) Olfactory abilities were measured by presenting a series of odors on cotton-tipped swabs into the test cage and subsequently scoring the videos of each session for number of seconds spent sniffing each swab. Statistical values represent genotype comparisons. Each odor-soaked swab was inserted into the cage for 2 minutes, in the sequence water, water, water, banana flavored, banana, banana, almond extract, almond, almond, soiled social cage 1, cage 1, cage 1, soiled social cage 2, cage 2, cage 2.

WT habituated to three presentations of the same odor, and dishabituated to the presentation of a new odor, in each case, for both males and females. *Shank3B* KO habituated to three presentations of the same odor, and dishabituated to the presentation of a new odor, in each case, for females. Male *Shank3B* displayed habituation and dishabituation to some odor presentations but not uniformly throughout the sequence. As combined male+female scores showed normal olfactory habituation/dishabituation for both genotypes on all odor presentations, and since sensory abnormalities appear in some cases of autism but are not diagnostic, the olfactory task was not repeated in Cohort 2. Bottom) Latency to first response on a 55°C hot plate did not differ between WT and *Shank3B* genotypes, in males, females, or combined males+females.

Table S10
Adult male-female reciprocal social interactions

	COHORT 1	COHORT 2
Male behavioral parameter		
Nose-to-anogenital sniffing time (sec)	t(1,22)=2.47, p<.03	t(1,18)=2.59, p<.02
Nose-to-anogenital sniffing bouts	t(1,22)=1.22, NS	t(1,18)=1.52, NS
Nose-to-nose sniffing time (sec)	t(1,22)=1.74, NS	t(1,18)=2.16, p<.05
Nose-to-nose sniffing bouts	t(1,22)=1.07, NS	t(1,18)=3.07, p<.01
Total sniffing time (sec)	t(1,22)=2.75, p<.02	*
Following time (sec)	t(1,22)=0.98, NS	t(1,19)=.177, NS
Following bouts	t(1,22)=1,18, NS	t(1,19)=.370, NS
Social contact time (sec)	t(1,22)=2.12, p<.05	*
Social contact bouts	t(1,22)=0.49, NS	*
Ultrasonic vocalizations (total number of calls)	t(1,22)=2.52, p<.02	t(1,18)=3.00, p<.01
Ultrasonic vocalizations (calls per minute)	F(1,44)=3.70, p<.01	F(1,19)=7.00, p<.05

Table S10. Male-female reciprocal social interactions. Genotype comparison of parameters of nose-to-anogenital sniffing and nose-to-nose sniffing indicated significant reductions or trends for less interactions in each cohort. Number of ultrasonic vocalizations emitted during the five minute test session were lower in both cohorts, as calculated in one minute time bins and as totals across the five minutes. Statistical values represent genotype comparisons. Previous work indicated that the source of vocalizations during male-female interactions is primarily from the males, although it remains possible that females emit a small number of calls. Genotype differences were not detected in all parameters in both cohorts. However, significant and trending indicators support an interpretation of reciprocal social interaction deficits in male *Shank3B* null mutants. Further replications of these findings will be useful.

*not scored in Cohort 2

Table S11
Contextual and cued fear conditioning

	COHORT 1
a) Pre-test Day 1	
Males	F(1,22)=3.59, NS
Females	F(1,20)=.239, NS
Combined	F(1,44)=1.32, NS
b) Training Day 1	
Males	F(1,22)=11.9, p<01
Females	F(1,20)=1.73, NS
Combined	F(1,44)=10.2, p<.01
c) Contextual Day 2	
Males	F(1,22)=.706, NS
Females	F(1,20)=3.55, NS
Combined	F(1,44)=3.67, NS
d) Pre-cue Day 3	
Males	F(1,22)=1.75, NS
Females	F(1,20)=.095, NS
Combined	F(1,44)=1.22, NS
d) Cued Day 3	
Males	F(1,22)=1.98, NS
Females	F(1,20)=2.03, NS
Combined	F(1,44)=3.45, NS

Table S11. Contextual and cued fear conditioning showed no genotype differences between *Shank3B* and WT in males, females, or combined males+females. However, during the training day, *Shank3B* males displayed significantly more freezing to the footshock than WT males. Statistical values represent genotype comparisons.

Table S12.
Morris water maze acquisition and probe trial

Acquisition

	COHORT 1
Training Day 1	
Males	F(1,14)=.910, NS
Females	F(1,18)=.509, NS
Combined	F(1,34)=1.75, NS
Training Day 2	
Males	F(1,14)=.064, NS
Females	F(1,18)=1.65, NS
Combined	F(1,34)=1.41, NS
Training Day 3	
Males	F(1,14)=2.09, NS
Females	F(1,18)=.010, NS
Combined	F(1,34)=1.08, NS
Training Day 4	
Males	F(1,14)=.208, NS
Females	F(1,18)=.020, NS
Combined	F(1,34)=.058, NS
Training Day 5	
Males	F(1,14)=.054, NS
Females	F(1,18)=2.44, NS
Combined	F(1,34)=.818, NS
Training Day 6	
Males	F(1,14)=4.87, p<.05
Females	F(1,18)=.182, NS
Combined	F(1,34)=2.08, NS
Training Day 7	
Males	F(1,14)=.480, NS
Females	F(1,18)=7.50, p<.05
Combined	F(1,34)=7.23, p<.05

Probe trial

	COHORT 1	
		Selective quadrant search
WT Males	F(1,9)=6.08, p<.01	Yes
WT Females	F(1,11)=6.39, p<.01	Yes
WT Combined	F(1,21)=12.8, p<.001	Yes

Shank3B Males	F(1,4)=3.88, p<.05	Yes
Shank3B Females	F1,7)=1.62, NS	No
Shank3B Combined	F(1,12)=1.12, NS	No

Table S12. Both genotypes and both sexes displayed learning of the hidden platform location across training trials in the Morris water maze. Using Repeated Measures ANOVA, overall latencies differed between genotypes across the learning curve, $F(1,34)=4.51$, $p<.05$. Posthoc analysis showed slower latencies to reach the platform on training day 6 in *Shank3B* males as compared to WT males, and on training day 7 in *Shank3B* females as compared to WT females. Statistical values represent genotype comparisons. Probe trial analysis for selective quadrant search time, defined as more time spent in the trained quadrant than in the other three quadrants, revealed that WT males, WT females, and *Shank3B* males displayed selective quadrant search. *Shank3B* females failed the probe trial. Combined scores indicated absence of selective quadrant search during the probe trial in *Shank3B*.
