# Supplementary material for: Replicable in vivo physiological and behavioral phenotypes of the Shank3B null mutant mouse model of autism
Source: Mol Autism. 2017 Jun 15;8:26. doi: 10.1186/s13229-017-0142-z (PMC5472997; doi:10.1186/s13229-017-0142-z)
Supplement: Supplementary file 2 — EEG low frequency power spectral analysis. (PDF 59 kb) [file 13229_2017_142_MOESM2_ESM.pdf]

Figure S2

EEG low frequency power spectral analysis

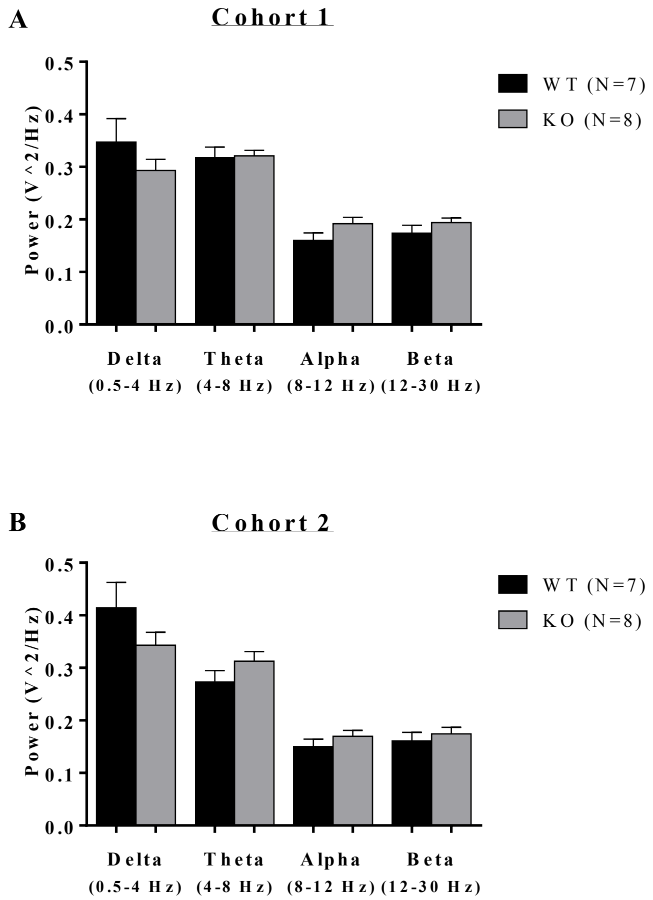

Figure S2. EEG low frequency power spectral analysis.

Spectral analysis of the one hour pre-PTZ EEG exhibits similar power in the delta (0.5-4 Hz), theta (4-8 Hz), alpha (8-12 Hz), and beta (12-30 Hz) frequency bands in Shank3B knockout (KO) relative to wildtypes (WT). These results were replicable in both cohorts. (A) Cohort 1, (B) Cohort 2.
